# Comparative Profiling of microRNA Expression in Soybean Seeds from Genetically Modified Plants and their Near-Isogenic Parental Lines

**DOI:** 10.1371/journal.pone.0155896

**Published:** 2016-05-23

**Authors:** Yong Wang, Qingkuo Lan, Xin Zhao, Wentao Xu, Feiwu Li, Qinying Wang, Rui Chen

**Affiliations:** 1 College of Plant Protection, Agricultural University of Hebei, Baoding, 071001, China; 2 Tianjin Institute of Agricultural Quality Standard and Testing Technology, Tianjin Academy of Agricultural Sciences, Tianjin, 300381, China; 3 College of Food Science and Nutritional Engineering, China Agricultural University, Beijing, 100083, China; 4 Institute of Agricultural Biotechnology, Jilin Academy of Agricultural Sciences, Changchun, 130033, China; Huazhong University of Science and Technology, CHINA

## Abstract

MicroRNAs (miRNAs) have been widely demonstrated to play fundamental roles in gene regulation in most eukaryotes. To date, there has been no study describing the miRNA composition in genetically modified organisms (GMOs). In this study, small RNAs from dry seeds of two GM soybean lines and their parental cultivars were investigated using deep sequencing technology and bioinformatic approaches. As a result, several differentially expressed gma-miRNAs were found between the GM and non-GM soybeans. Meanwhile, more differentially expressed gma-miRNAs were identified between distantly relatednon-GM soybeans, indicating that the miRNA components of soybean seeds varied among different soybean lines, including the GM and non-GM soybeans, and the extent of difference might be related to their genetic relationship. Additionally, fourteen novel gma-miRNA candidates were predicted in soybean seeds including a potential bidirectionally transcribed miRNA family with two genomic loci (gma-miR-N1). Our findings firstly provided useful data for miRNA composition in edible GM crops and also provided valuable information for soybean miRNA research.

## Introduction

MicroRNAs (miRNAs) are a class of genome-encoded, single-stranded, hairpin precursor-derived, small non-coding RNAs that can regulate gene expression at the transcriptional and post-transcriptional levels [1]. Because of their fundamental roles and common features, miRNAs have attracted considerable attention and a great deal of research has been reported in this field in recent years. To date, 35,828 mature miRNAs and 28,645 stem-loop precursors from 223 species including plants, animals, viruses, and even single-celled organisms have been identified and registered in miRBase (Release 21.0) [2]. In the medical field, circulating extracellular miRNAs are used as clinical biomarkers in disease prevention and diagnosis [3, 4]. Due to their endogeneity and high regulatory specificity, the research of miRNAs has resulted in great progress in many medical fields, such as cancer therapy, small RNA medicine, and the treatment of viral disease [5–7]. Further studies on the biogenesis and functions of miRNAs have revealed that the universality and complexity of miRNAs far exceed our current understanding, and additional studies are needed to refine the roles of small RNA and expand our knowledge of gene expression and regulation [8–10].

The introduction of genetically modified (GM) crops has led to significant increases in agricultural output by enhancing plant tolerance to biotic or environmental stress in the past few decades. According to a statistical analysis, the global area of GM cultivars increased to more than 175 million hectares in 2013 [11]. Soybean (*Glycine max*) is one of the most important economic crops for protein and oil supplements in the world, and it is a major target for transgenic modification. At present, several GM soybean lines have been developed and approved for commercialization. Insect resistance and herbicide tolerance are the main agronomic traits that have been improved in GM soybean [12]. With the rapid increase in the area used for GM crops, more attention has been focused on security issues related to the impact of genetically modified organisms (GMOs) on the environment and food safety for the public. To date, more than 50 countries and areas have established separate legislation and regulations for labeling foods that contain GM ingredients [13]. Although there are only a few reports claiming that GM crops could have negative consequences [14–16], research assessing the potential risks associated with GMOs should continue.

Cross-kingdom regulation of gene expression by miRNA was first discovered and reported by Zhang *et al*. in 2012 [17]. Experiments performed *in vivo* and *in vitro* indicated that rice miR168a could be found in the human blood circulatory system and could inhibit a mammalian gene expression (low-density lipoprotein receptor adapter protein 1, LDLRAP1). Compared to past work focusing on intracellular and intercellular regulation by miRNAs, the discovery of cross-kingdom regulation by miRNAs provided strong evidence for the co-evolving relationship between humans and edible plants, whereby miRNAs play key roles in information exchange at the molecular level. Furthermore, the discovery of cross-kingdom regulation also raised new concerns regarding miRNA components in GM crops.

As with messenger RNAs (mRNAs) in plants and animals, the expression of some miRNAs is variable between tissues or developmental stages, particularly in organisms under stress[18–21]. Considering that transgenic manipulation involves inserting foreign genes or modifying the activity of existing genes, it is reasonable to propose that certain endogenous miRNAs found in GMOs might be affected and differentially expressed. Taking advantage of high-throughput sequencing technology, it is feasible to accurately evaluate the miRNA composition of individual organisms. On the other hand, RNA stored in dry seeds was shown to be relatively stable, and soybean seeds are directly edible after processing, for example as soybean milk [22], which is of practical significance for further research about potential functions for plant miRNAs in human body. Based on these considerations, the dry seeds of GM plants and their parental cultivars were selected for this study.

The current evaluation system for GM crops is focused on proteins, fats, carbohydrates, toxins, and nutritional ingredients. However, miRNAs have not previously been taken into account. Herein, four small RNA libraries were constructed from two GM soybeans (MON89788 and DP-3Ø5423×GTS 40-3-2) and their acceptor lines. After deep sequencing and bioinformatic analysis, several conserved gma-miRNAs were found to be differentially expressed in dry seeds. Combined with miRcheck prediction and experimental verification, fourteen novel gma-miRNA candidates and their stem-loop precursors were also identified [23]. Interestingly, a large proportion of small RNAs generated from ribosomal RNA genes in chromosome Gm13 in soybean seeds was also discovered. Taken together, these results provided useful information about the miRNA composition in GM crops and this work contributes to an improved understanding of miRNAs in soybean seeds.

## Materials and Methods

### GM soybean samples and RNA isolation

The transgenic soybean line MON89788 and non-GM soybean A3244 were provided by the Qinhuangdao Entry-Exit Inspection and Quarantine Bureau (Qinhuangdao, China). Stacked GM soybean DP-3Ø5423×GTS 40-3-2 and its non-GM counterpart Jack were provided by DuPont Company (USA). Conventional PCR experiments were performed with event-specific primers for verification of the GM soybeans. For each soybean line, three biological replicates were used. For each replicate sample, three aliquots (~ 50 mg each) were randomly drawn and subjected to total RNA extraction. Nine RNA samples were combined for construction of small RNA libraries.

An improved SDS/guanidine isothiocyanate method was used for total RNA isolation from soybean seeds. The details of the procedure are listed as follows. One milliliter of SDS lysis buffer (100 mM NaAc-HAc pH 5.2; 10 mM EDTA; 200 mMNaCl; 1% SDS; 2% PVPP; 1% 2-mercaptoethanol) was added to 50 mg powder and vortexed vigorously. The samples were incubated at 60°C for 10 min, and a one-third volume of 5 M KAc (pH 4.8) was added. The tube was vortexed, placed in ice water for 10 min and then centrifuged for 15 min at 13,000×g at 4°C. The supernatant was transferred to a new tube, and an equal volume of RNA extraction buffer (38% v/v phenol in DEPC-H_2_O, 1 M guanidinium isothiocyanate, 1 M ammonium thiocyanate, 0.1 M sodium acetate pH 5.0) was added. The tube was vortexed for 3 min and placed at room temperature for 5 min. Then, the samples were extracted once with chloroform:isoamyl alcohol (24:1) and twice with acid phenol:chloroform:isoamyl alcohol (25:24:1). A one-tenth volume of 3 M NaAc (pH 5.0) and an equal volume of ice-cold isopropanol were added. The tube was held at room temperature for 10 min and centrifuged for 15 min at 12,000×g at 4°C. The pellet was washed twice with 75% ethanol, and the RNA was dissolved in DEPC water. The quantity and quality of total RNA were evaluated using a NanoDrop 1000 Spectrophotometer and 1% (w/v) agarose gel electrophoresis.

### Small RNA library construction

The small RNA libraries were constructed using the TruSeq Small RNA Sample Preparation kit according to the manufacturer’s instructions. The cDNA products were checked for their quantity and quality using the Agilent 2100 BioAnalyzer and deep sequenced using Illumina/Solexa HiSeq 2000 platform. The raw data from the small RNA libraries were deposited at the NCBI Sequence Read Archive (SRA) under accession no. SRP051931.

### Bioinformatic analysis of high-throughput data

For preprocessing the high-throughput data, the raw reads were first screened according to the sequence quality, and individual reads with more than four Phred scores below 15 were discarded. The high-quality reads were then processed by trimming the adaptor sequence, consolidating the identical reads and recording the abundance. Only reads with a reliable 3’ adaptor tail and no ‘N’ or adaptor contaminants were used to generate the clean reads. The soybean (*Glycine max*) genome and annotation data were downloaded from the plantGDB database[24]. SOAP (v1.11) was used for genome mapping, and only perfectly matched reads were subjected to further analysis [25]. Customized Perl scripts were designed and written to annotate the small RNA loci and analyze their chromosomal distribution.

### Differential expression analysis of conserved gma-miRNAs

Known soybean miRNAs (gma-miRNAs) and their stem-loop precursor sequences (gma-MIRs) were downloaded from miRBase [2]. For differential expression analysis, the abundance of each gma-miRNA was normalized to the number of total clean reads in each small RNA library. The P-value was calculated using the formula given below based on previous reports [26, 27]. *N*_*1*_ and *N*_*2*_ are the total numbers of clean reads in the non-GM and GM soybean libraries, and *x* and *y* are the numbers of gma-miRNA reads in the non-GM and GM soybean libraries, respectively. The log_2_ ratio was calculated as log_2_(number of gma-miRNA reads in GM soybean/number of gma-miRNA reads in non-GM soybean). The Winflat program was integrated into Perl scripts for statistical calculation of the P-value [26].

$$p(x|y)={\left(\frac{N_{2}}{N_{1}}\right)}^{y}\frac{(x+y)!}{x!y!{\left(1+\frac{N_{2}}{N_{1}}\right)}^{\left(x+y+1\right)}}$$

$$p=min\left\{\sum_{k=0}^{k\leq y}p(k|x),\sum_{k=y}^{\infty }p(k|x)\right\}$$

### Novel miRNA prediction

For novel miRNA prediction, the clean reads in the four libraries were combined and BLAST searched against the Rfam database to remove known non-coding RNAs [28]. The RNAfold and miRCheck programs were utilized for stem-loop structure determination and novel miRNA prediction with the default parameters [23]. The details of this process were described in our previous work [29]. The only difference was that double strand testing step was omitted. psRNATarget online was used for target prediction of novel miRNA candidates [30].

### RT-PCR assays

Differential expression of conserved gma-miRNAs was verified by quantitative RT-PCR experiments. One microgram of total RNA was reverse-transcribed and amplified using miRcute miRNA First-Strand cDNA synthesis kit (Tiangen, KR201) and miRcute miRNA qPCR detection kit (Tiangen, FP401). The comparative ΔΔCT method was used for relative quantitation of these gma-miRNAs [31]. The U6 snRNA was selected as the endogenous reference gene for normalization. For validation of novel gma-miRNA candidates, a diluted cDNA template was used for RT-PCR assays with specific forward primers and the universal reverse primer. The amplified products were detected by 2% (w/v) agarose gel electrophoresis and validated by Sanger sequencing. The primers used in this section are listed in **S3 Table**.

## Results

### Overview of small RNA libraries

**Fig 1:**
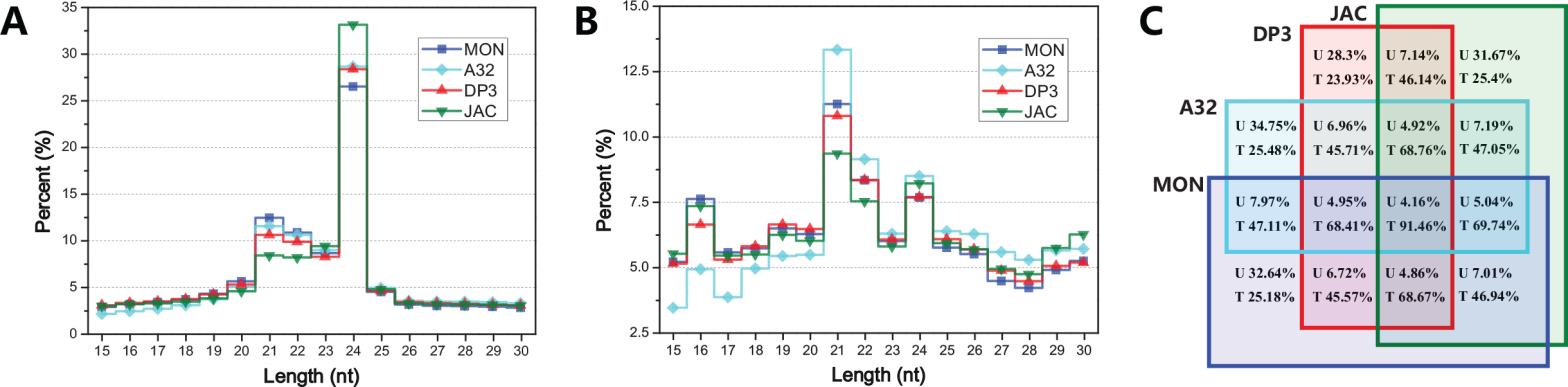
Length distribution and Venn diagram of small RNA libraries. A: Length distribution of the unique reads. B: Length distribution of the total reads. C: Venn diagram of four small RNA libraries. All of the small RNA reads from four libraries are combined as whole. Each percentage is calculated by dividing the number of reads in each library. U, unique; T, total; MON, MON89788; A32, A3244; DP3, DP-3Ø5423×GTS 40-3-2; JAC, Jack.

Four small RNA libraries from MON89788, A3244, DP-3Ø5423×GTS 40-3-2 and Jack soybean seeds generated 36,474,511, 36,937,578, 33,667,451 and 40,500,433 raw reads using a deep sequencing platform. After preprocessing, more than 18 million clean reads, corresponding to 1.3~1.6 million unique reads, were obtained from each library. Approximately 17 million clean reads corresponded to at least one perfectly matching locus in the soybean genome, and about 82% of them were located in intergenic regions (**Table 1**). According to the annotation information, approximately 3 million reads were located in genes or transcripts, and the proportions of exonic and intronic sequences were nearly equal. Intriguingly, a high-density region for small RNA generation was discovered on chromosome Gm13. More than three quarters of the total reads in each library were perfectly matched to a 1 Mb fragment (spanning from 14.7 M to 15.7 M of Gm13) with obvious strand bias (**S1 Fig**). Further analysis of this genomic locus showed that it’s a super tandem repeat of ribosomal RNA genes, including more than 100 rRNA genes, which was not annotated correctly in Phytozome. A previous report had described the rRNA-derived small RNAs occupying approximately 10% in a soybean small RNA library from germinating cotyledons [32]. However, such a large amount of rRNA-derived small RNAs (~75%) in soybean seeds had never been discovered before. The high proportion of ribosomal related small RNAs as storage ingredient in soybean seeds suggested that they might be functional and play crucial roles during seed germination. As shown in **Fig 1A** and **1B**, 21-nt and 24-nt classes of clean reads represented the majority of the unique and total reads, respectively. There was no obvious difference in the length distribution between the GM and non-GM libraries. In contrast to the 21-nt class, small RNAs in 24-nt class showed more polymorphism. Common read analysis indicated that fewer shared reads were found when more libraries were included. Only 4.16% of the unique reads were shared among the four libraries, accounting for 91.46% of the total reads (**Fig 1C**).

**Table 1:** Composition of small RNA libraries from soybean seeds of GM and their parental lines.

			MON		A32		DP3		JAC	
			Unique	Total	Unique	Total	Unique	Total	Unique	Total
Raw reads			—	36,474,511	—	36,937,578	—	33,667,451	—	40,500,433
High quality reads			—	34,851,791	—	35,306,825	—	32,211,029	—	38,797,935
Clean reads (15–30 nt)			1,509,201	19,281,725	1,607,094	19,515,644	1,308,765	18,327,863	1,464,627	19,452,871
Not matching genome			791,683	2,230,283	879,611	2,469,019	655,168	1,858,850	723,226	2,083,080
Matching genome			717,518	17,051,442	727,483	17,046,625	653,597	16,469,013	741,401	17,369,791
	Intergenic		—	14,215,639	—	14,025,360	—	13,571,051	—	14,410,600
	Gene (transcripts)		—	2,835,803	—	3,021,265	—	2,897,962	—	2,959,191
		Exon	—	1,639,965	—	1,811,152	—	1,713,607	—	1,741,190
		Intron	—	1,195,838	—	1,210,113	—	1,184,355	—	1,218,001
Matching gma-MIRs			2,655	625,399	2,649	1,103,604	2,562	742,318	2,712	613,667
	Corresponding gma-MIRs		386	—	362	—	360	—	375	—
	Mature gma-miRNAs		144	491,705	153	958,430	139	621,249	138	492,651
Matching Rfam database			33,200	2,126,950	33,153	1,964,365	32,117	2,044,085	33,354	2,246,844
	rRNA		12,381	1,783,876	12,719	1,643,676	12,608	1,726,000	12,665	1,895,127
	tRNA		2,069	199,903	2,115	194,209	1,828	191,841	2,017	221,421
	snoRNA		8,365	71,627	7,247	56,350	6,477	47,812	7,691	55,875
	other		10,385	71,544	11,072	70,130	11,204	78,432	10,981	74,421
Unknown Reads			681,663	14,299,093	691,681	13,978,656	618,918	13,682,610	705,335	14,509,280

MON, MON89788; A32, A3244; DP3, DP-3Ø5423×GTS 40-3-2; JAC, Jack.

### Differential expression analysis of conserved gma-miRNA

**Fig 2:**
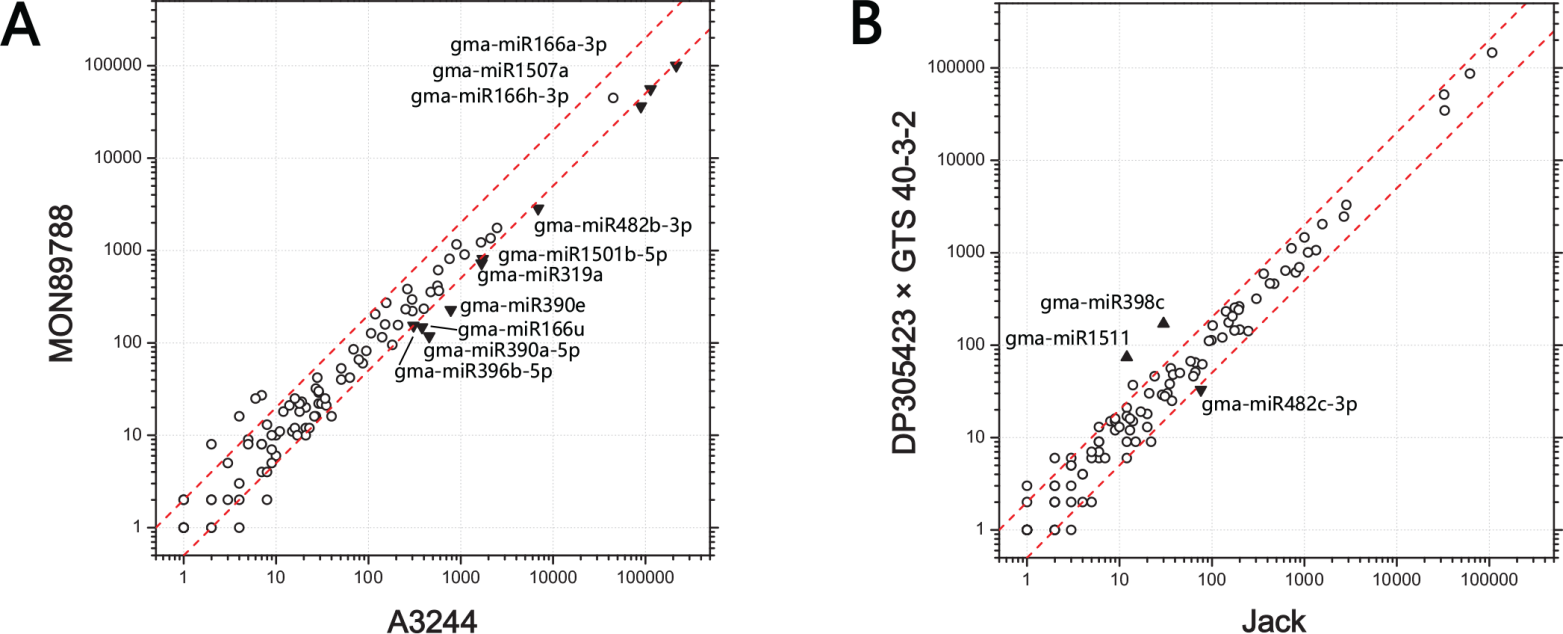
Scatter plot demonstrating the differential expression of gma-miRNAs in GM soybean seeds. The normalized values were calculated using the following formula: (miRNA reads count/total clean reads) × 10^7^. Red dotted lines indicate the value of |log_2_| = 1. Down- or up-regulated gma-miRNAs are indicated with black triangles; these gma-miRNAs meet the following three conditions: raw reads of gma-miRNAs > 100, |log_2_ ratio| > 1, and P < 0.001. A: Results of the comparison between MON89788 and A3244. B: Results of the comparison between DP-3Ø5423×GTS 40-3-2 and Jack.

To detect conserved gma-miRNAs, the clean reads were aligned against known gma-miRNAs represented in miRBase. Only identical reads were regarded as mature gma-miRNAs. In total, at least 138 gma-miRNAs were found in each small RNA library. Meanwhile, more than 380 gma-MIR precursors could find perfectly matching reads in each library (**S1 Table**). For differential profiling expression analysis, the read numbers were normalized to calculate the P-values and log_2_ ratios. Differences in expression were considered significant at P < 0.001 and a log_2_ ratio > 1. Totally, three couples were analyzed, including MON89788 and A3244, DP-3Ø5423×GTS 40-3-2 and Jack, A3244 and Jack. As a result, ten gma-miRNAs were down-regulated in MON89788 compared with A3244 soybeans, including mature members of the miR1507, miR1510, miR166, miR319, miR390, miR396 and miR482 families. In DP-3Ø5423×GTS 40-3-2 and Jack soybeans, only miR482c-3p was down-regulated, and two gma-miRNAs, miR398c and miR1511, were up-regulated. Between non-GM soybneas, A3244 and Jack, thirteen varied gma-miRNAs were also identified where ten gma-miRNAs were up-regulated and three gma-miRNAs were down-regulated (**Fig 2**).

**Fig 3:**
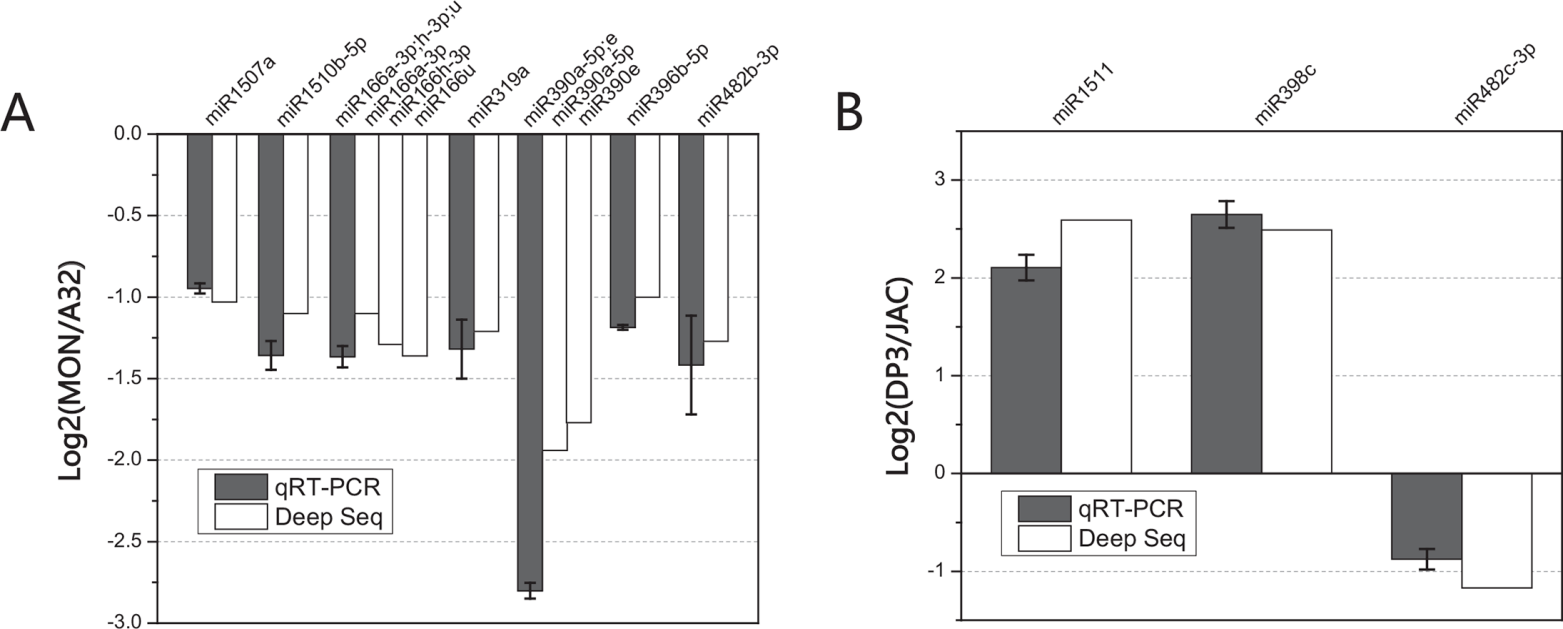
Verification of differentially expressed gma-miRNAs by quantitative RT-PCR. Identical primers were designed for similar mature miRNAs. The comparative ΔΔCT method was used for the quantitative RT-PCR experiments and the U6 snRNA was selected as the reference gene. A: Results of the comparison between MON89788 and A3244. B: Results of the comparison between DP-3Ø5423×GTS 40-3-2 and Jack.

The maximum values of log_2_ ratio for up- and down-regulation were 2.79 and -1.94 for miR398c and miR390a-5p, respectively (**Table 2**). With regard to abundance, gma-miR166a, gma-miR166h and gma-miR1507a were the most abundant miRNAs among four small RNA libraries, with 70 ~ 420 thousand clean reads before normalization (**S2 Fig**). These results indicated that differences of miRNA composition exist between GM soybean seeds and their parental non-GM lines. Meanwhile, differentially expressed miRNAs could also be found between non-GM soybeans, which might be more diverse due to their distant genetic relationship. Although we could not determine the intrinsic factors responsible for these differences, it seemed that differentially expressed miRNAs in soybean seeds are ubiquitous between different cultivars including the GM and non-GM soybeans. Additionally, thirteen differentially expressed gma-miRNAs identified by deep sequencing, two were up-regulated and eleven were down-regulated in GM soybean seeds compared with their parental lines, were confirmed by quantitative real-time PCR (**Fig 3**).

**Table 2:** Differential expression of known gma-miRNAs in soybean seeds.

Sequence (5’-3’)	Mature miRNA	Log2(MON/A32)		Log2(DP3/JAC)		Log2(A32/JAC)		Same to gma-miRNAs
TCTCATTCCATACATCGTCTGA	miR1507a	-1.03	↓	0.49	—	0.88	—	gma-miR1507a
AGGGATAGGTAAAACAACTACT	miR1510b-5p	-1.10	↓	-0.34	—	0.98	—	gma-miR1510b-5p
AACCAGGCTCTGATACCATG	miR1511	0.06	—	2.59	↑	2.07	↑	gma-miR1511
TTGACAGAAGATAGAGAGCAC	miR156c	-0.44	—	0.70	—	1.07	↑	gma-miR156c,d,e,i,j,l,m
TCGGACCAGGCTTCATTCCCC	miR166a-3p	-1.10	↓	0.44	—	1.00	↑	gma-miR166a-3p,b,c-3p,d,e,f,g,n,o,i-3p
TCTCGGACCAGGCTTCATTCC	miR166h-3p	-1.29	↓	0.66	—	1.46	↑	gma-miR166h-3p,k
TCTCGGACCAGGCTTCATTC	miR166u	-1.36	↓	0.41	—	0.95	—	gma-miR166u
GGAGATGGGAGGGTCGGTAAAG	miR2118a-5p	-0.38	—	-0.46	—	-1.10	↓	gma-miR2118a-5p,b-5p
TTGGACTGAAGGGAGCTCCC	miR319a	-1.21	↓	0.62	—	1.21	↑	gma-miR319a,b,e
AAGCTCAGGAGGGATAGCGCC	miR390a-5p	-1.94	↓	0.21	—	1.58	↑	gma-miR390a-5p,f,g
AGCTCAGGAGGGATAGCGCC	miR390e	-1.77	↓	0.31	—	2.00	↑	gma-miR390e
TTCCACAGCTTTCTTGAACTT	miR396b-5p	-1.00	↓	0.30	—	0.88	—	gma-miR396b-5p,c
TGTGTTCTCAGGTCGCCCCTG	miR398c	-0.42	—	2.49	↑	2.79	↑	gma-miR398c
TGTTGCGGGTATCTTTGCCTC	miR4412-5p	0.36	—	-0.12	—	-1.57	↓	gma-miR4412-5p
TCTTCCCTACACCTCCCATACC	miR482b-3p	-1.27	↓	0.21	—	1.27	↑	gma-miR482b-3p,d-3p
TTCCCAATTCCGCCCATTCCT	miR482c-3p	-0.93	—	-1.17	↓	1.26	↑	gma-miR482c-3p
TGAAGATTTGAAGAATTTGGGA	miR5376	-0.46	—	-0.36	—	-1.19	↓	gma-miR5376

MON, MON89788; A32, A3244; DP3, DP-3Ø5423×GTS 40-3-2; JAC, Jack. gma-miRNAs with original abundance more than 100 reads were listed. Log_2_ ratios of normalized gma-miRNA reads from GM and non-GM soybean seeds were calculated, P < 0.001.

### Novel gma-miRNA prediction

**Fig 4:**
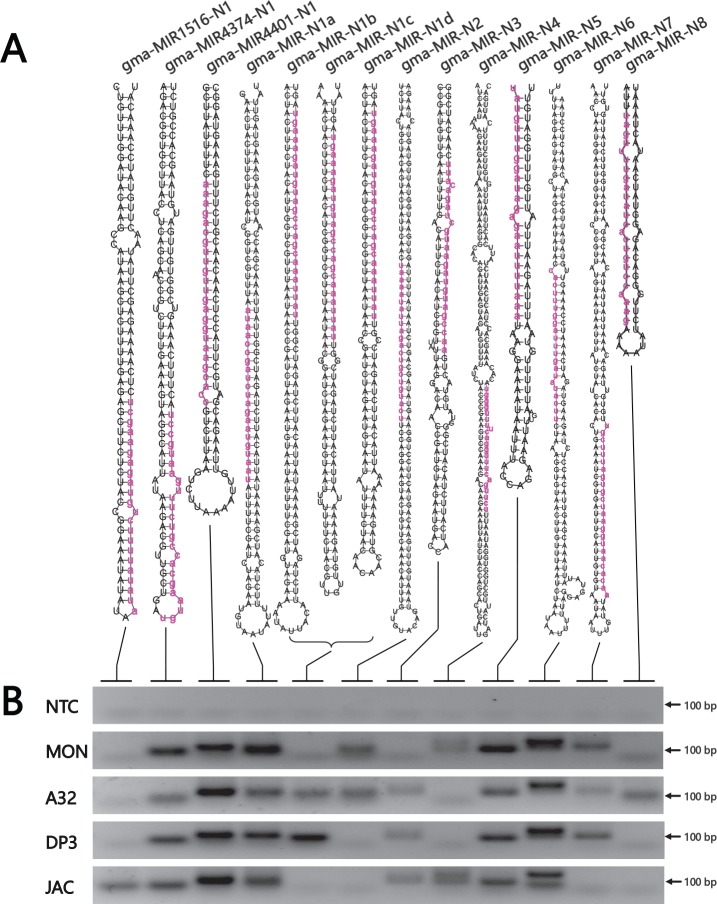
Novel gma-miRNA candidates found in this study. A: Hairpin structures of novel gma-miRNA precursors predicted in GM soybean seeds. Mature miRNAs are indicated with red lowercase letters. B: Expression analysis of novel gma-miRNA candidates by RT-PCR. NTC, no template control; MON, MON89788; A32, A3244; DP3, DP-3Ø5423×GTS 40-3-2; JAC, Jack.

According to the standards of plant miRNA prediction, the miRCheck program was employed to investigate novel miRNAs in soybean. Soybean miRNAs have been extensively surveyed, and 639 mature miRNAs corresponding to 573 precursors have been registered in miRBase. As a result, only fourteen gma-miRNA candidates were finally acquired with relatively low abundance levels based on our optimal pipelines with strict parameters. Among these candidates, 3 stem-loop precursor sequences shared high similarity to the known gma-MIRs, gma-MIR1516a, gma-MIR4374b and gma-MIR4401a (**S3 Fig**). However, the most abundant reads of these three conserved gma-MIRs were not identical to known gma-miRNAs. The other eleven miRNA precursors were capable to form characteristic hairpin structures without any homology to known gma-MIRs. Notably, gma-miR-N1 corresponded to a potential bidirectionally transcribed miRNA that was first reported in *Drosophila* [33]. Four members of the gma-miR-N1 family were most likely produced from both the sense and the antisense strands of two genomic loci (**S4 Fig**). For all of the newly identified gma-miRNAs, the preferred nucleotides at the 5’ end were uracil and adenine, and most of these miRNAs were 24 nt in length. All novel gma-miRNA candidates were subjected to experimental verification by RT-PCR and Sanger sequencing, and they all were positively identified in at least one library (**Fig 4, Table 3 and S1 File**). Target prediction was performed using psRNATarget online for the novel gma-miRNA candidates [30]. Based on the available annotation information, the target genes of gma-miR-N4 and gma-miR-N5 are an integral membrane protein and the BZIP transcription factor. The results are listed in **S2 Table**.

**Table 3:** Results of novel gma-miRNA prediction in soybean seeds.

No.	Mature miRNAs	Sequence(5’-3’)	Length	Clean reads in each library					Precursor location	Precursor Conservativeness
				Total	MON	A32	DP3	JAC		
1	gma-miR1516-N1	AUAUAUUUUCUGUAGAGAAGCU	22 nt	5	0	0	2	3	Gm03_-_47035754_47035862	gma-MIR1516a
2	gma-miR4374-N1	UGUAAGCACCGUCUUUGAAUGCCU	24 nt	42	18	8	9	7	Gm01_-_23428261_23428375	gma-MIR4374b
3	gma-miR4401-N1	AAAGACGUUGUUGAGGUAAGCACC	24 nt	6	3	2	0	1	Gm05_-_5369728_5369821	gma-MIR4401a
4	gma-miR-N1a	AUAACCGAUCUAGAAUGUAAU	21 nt	10	3	5	0	2	Gm02_+_39925524_39925657	None
5	gma-miR-N1b	UAUUAAACGACCGAUGUAGAAAGU	24 nt	13	1	1	10	1	Gm02_-_39925526_39925654	None
6	gma-miR-N1c	UAUUAAACGACCGAUGUAGAAAGU	24 nt	13	1	1	10	1	Gm03_+_11919586_11919706	None
7	gma-miR-N1d	UAUUAAACGACCGAUGUAGAAAGU	24 nt	13	1	1	10	1	Gm03_-_11919589_11919703	None
8	gma-miR-N2	UAAAAUAUUAGACUGCUGUACU	22 nt	68	37	24	4	3	Gm06_-_46035186_46035339	None
9	gma-miR-N3	AACCGAUGUAGAAUGCUAGACAUU	24 nt	25	4	2	5	14	Gm07_-_19042117_19042232	None
10	gma-miR-N4	UCUUGACUUUGGACUUUUGGGU	22 nt	20	4	7	1	8	Gm02_-_48422489_48422679	None
11	gma-miR-N5	UAUGUUUGGAUAGAGAAUUUUAAA	24 nt	11	3	5	0	3	Gm07_+_44428050_44428136	None
12	gma-miR-N6	CACUUUGGAUUUGAUAUACUU	21 nt	6	1	4	0	1	Gm05_+_40112942_40113095	None
13	gma-miR-N7	AACACAAUGGAAUCGUGAUUUCGU	24 nt	5	1	2	0	2	Gm07_+_1788665_1788817	None
14	gma-miR-N8	UAGUUUUGAUAUCACUGUCCAAAG	24 nt	5	1	3	0	1	Gm08_-_4271026_4271087	None

MON, MON89788; A32, A3244; DP3, DP-3Ø5423×GTS 40-3-2; JAC, Jack.

## Discussion

MicroRNAs have been a topic of intense interest in current molecular biology, and their biogenesis and functions have been thoroughly studied over the past decade. Nevertheless, no studies have been reported comparing the miRNA composition between GM crops and their near-isogenic lines prior to the discovery of phenomenon of cross-kingdom regulation. Evidence that rice miR168 could enter the human bloodstream and regulate mammalian gene expression has expanded our understanding of miRNA [17]. Although many subsequent reports have questioned this controversial result [34–37], the findings offer a novel perspective for miRNA research. The purpose of this study was to identify the differentially expressed miRNAs in GM soybean seeds using deep sequencing technology and to provide data that will be useful for the future research.

Soybean is one of the most important natural sources for dietary oil and proteins worldwide. The choice to use the dry seeds of GM soybeans for this study was mainly based on the consideration that soybean can be directly consumed as a food, for example as soybean milk, which makes it practical for future research on cross-kingdom regulation of plant miRNA. The MON89788 GM soybean is a genetically engineered variant of the near-isogenic conventional soybean variety A3244. A *cp4 EPSPS* gene with stronger constitutive viral promoters was introduced via Agrobacterium-mediated transformation to improve the glyphosate tolerance of the plant [38]. The stacked GM soybean DP-3Ø5423×GTS 40-3-2 has the same genetic background as the non-GM Jack soybean except that it contains insertions of the endogenous genes, *gm-fad2-1*, *gm-hra* and *cp4*, which were introduced through crossbreeding with the high oleic acid soybean DP-3Ø5423 and the Roundup Ready soybean 40-3-2. Subchronic feeding studies in rats have been reported using these GM soybeans, and no obvious quantitative differences were found in the proteins, amino acids, nutritional factors or systemic effects [39–41]. Although there were no obvious differences in the contents of biological macromolecules, it’s reasonable to believe that the sustained expression of exogenous genes *in vivo* might trigger unexpected interactions and alter compositional properties at the RNA level. So far, reports on the RNAs present in dormant soybean seeds are scarce, however the storage proteins, globulins, 7S β-conglycinin and 11S glycinin, have been heavily studied to characterize their patterns of accumulation and associated regulatory factors prior to dormancy [22, 42, 43]. Recent research on miRNAs in plant seeds has addressed their potential regulatory roles in miRNA-mediated gene expression during seed development and germination [44, 45]. However, no study has been performed to investigate differences in the composition of miRNAs in GM soybeans.

In this study, we identified a largely repeated rRNA gene region for small RNA production at the approximately 15 Mb locus on chromosome Gm 13. There is an obvious strand bias and the mapping ratio between sense and antisense strands is about 1:100. Unbalanced mapping depth along the rRNA gene indicates that they are authentic small RNAs instead of rRNA degradation products. Although these rRNA-derived small RNAs had been reported to be involved in various signaling pathways and have potential biological functions, the underlying mechanism is still largely unknown [46]. Similarly to storage proteins, these specific small RNAs might be integral components of soybean seeds that function as molecular regulators to control gene expression during seed germination like miRNA [47]. Further studies on this abundant class of rRNA-derived small RNAs in soybean seeds will provide new insights into seed biology.

For novel miRNA prediction, an optimal pipeline based on plant miRNA criteria and the miRCheck program described in our previous work was used [29, 48]. Considering that miRNAs are known to be tissue-, condition- and developmental stage-specific in many eukaryotes, the current set of registered miRNAs in miRBase might be only part of the complete set of authentic miRNAs in soybean. Although the gma-miRNAs are well annotated in domestic soybean, fourteen novel miRNA candidates were discovered in this study, including three members of known conserved gma-miRNA families. All the precursors of these novel miRNAs could form perfect hairpin structures, indicating that our bioinformatic pipeline is reliable and sensitive. A potential family of bidirectionally transcribed miRNAs was also identified with low abundance, suggesting the complexity and diversity of gma-miRNAs.

In this work, a deep sequencing platform was utilized for comparative profiling of miRNA expression in soybean seeds of GM plants and their near-isogenic acceptor lines. As expected, several conserved gma-miRNAs were found to be differentially expressed more than 2-fold change, and three conserved gma-miRNAs were legume-specific. Compared to A3244, all ten of the differentially expressed gma-miRNAs in MON89788 were down-regulated, including the members of miR1507, miR1510, miR166, miR319, miR390, miR396 and miR482 families. Two of the most abundant down-regulated miRNAs were gma-miR166a-3p and gma-miR166h-3p. miR166 has been reported to regulate the homeodomain leucine zipper (HD-ZIP) gene, which might play significant roles in seedling development [49]. Between DP-3Ø5423×GTS 40-3-2 and Jack soybeans, three varied gma-miRNAs were identified with low or moderate abundance where miR482c-3p was down-regulated, and miR398c and miR1511 were up-regulated. Notably, thirteen differentially expressed gma-miRNAs were also discovered when comparing non-GM soybeans (A3244 and Jack). These results implied that the miRNA components of soybean seeds varied among different soybean lines, and the extent of difference might be related to their genetic relationships. For a better understanding of these differentially expressed miRNAs, further research is required to investigate their functions and whether they could influence human gene expression due to uptake into the bloodstream upon consumption.

## Supporting Information

S1 Fig Chromosomal distribution of rRNA derived small RNAs. (PDF)

S2 Fig Statistics of conserved gma-miRNA families. (PDF)

S3 Fig Alignments of gma-miRNA precursors. (PDF)

S4 Fig Alignments between mature miRNAs and their presursors. (PDF)

S1 File Sanger sequencing results for novel miRNA candidates. (DOCX)

S1 Table Statistics of conserved gma-miRNAs found in this study. (XLSX)

S2 Table Target prediction of novel gma-miRNAs using psRNATarget online. (DOCX)

S3 Table Primers used for qRT-PCR and RT-PCR experiments. (DOCX)
