# Supplementary material for: Comparative Profiling of microRNA Expression in Soybean Seeds from Genetically Modified Plants and their Near-Isogenic Parental Lines
Source: PLoS One. 2016 May 23;11(5):e0155896. doi: 10.1371/journal.pone.0155896 (PMC4876996; doi:10.1371/journal.pone.0155896)

Supplemental File:
Figure_S1. Chromosomal distribution of rRNA derived small RNAs.

Paper title: "Comparative profiling of microRNA expression in soybean seeds from genetically modified plants and their near-isogenic parental lines"
Author: Yong Wang, Qingkuo Lan, Xin Zhao, Wentao Xu, Feiwu Li, Qinying Wang*, Rui Chen*
Date: Mar. 2016
Contact: chenrui.2011@outlook.com.

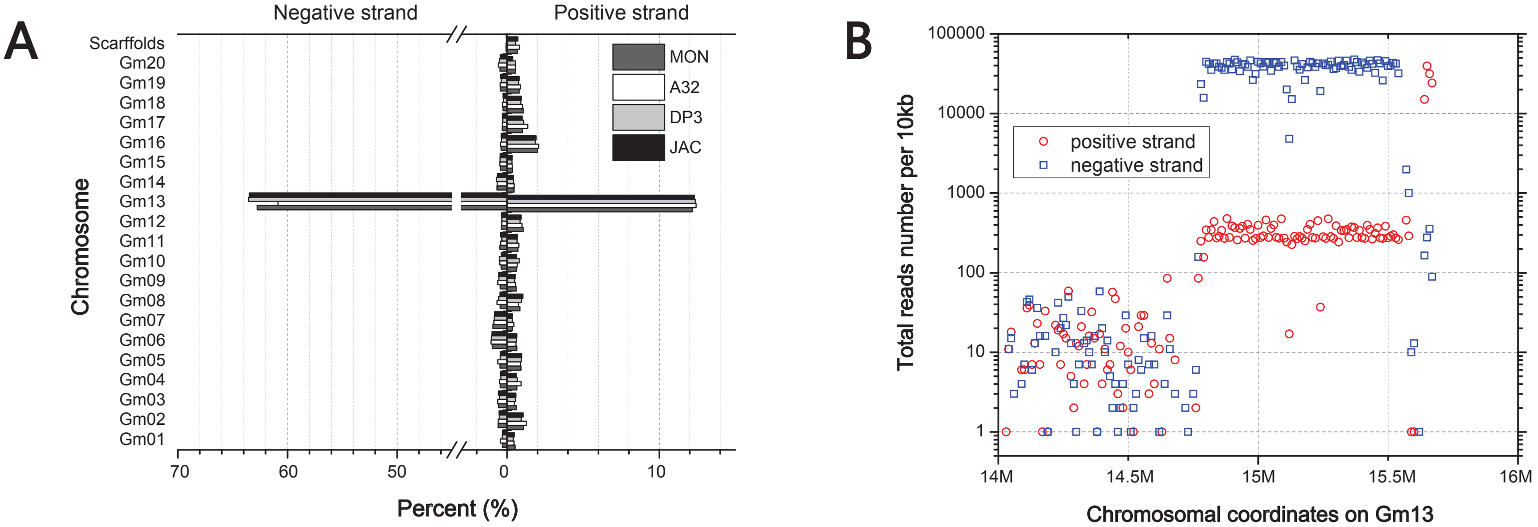

Supplement: S1 Fig — (PDF) [file pone.0155896.s001.pdf]
