# Supplementary material for: Comparative Profiling of microRNA Expression in Soybean Seeds from Genetically Modified Plants and their Near-Isogenic Parental Lines
Source: PLoS One. 2016 May 23;11(5):e0155896. doi: 10.1371/journal.pone.0155896 (PMC4876996; doi:10.1371/journal.pone.0155896)
Supplement: S4 Fig — (PDF) [file pone.0155896.s004.pdf]

Supplemental File:
Figure_S4. Alignments between mature miRNAs and their presursors.

Paper title: "Comparative profiling of microRNA expression in soybean seeds from genetically modified plants and their near-isogenic parental lines"

Author: Yong Wang, Qingkuo Lan, Xin Zhao, Wentao Xu, Feiwu Li, Qinying Wang*, Rui Chen*

Date: Mar. 2016

Contact: chenrui.2011@outlook.com

################################

3 conserved gma-MIRs alnout:

```
gma-MIR1516-N1
>F_20_01_Gm03_-_start_47035754_stop_47035862_length_109nt mappable_miRNA_candidates_9      gma-MIR1516a  89.9   I109MI
CUGUUUGGAUACAAGCCAUAAGCUCUUUUGAGAGCUUCUCUACCGGAAAUAUAUAAUAUAUUUUCUGUAGAGAAGCUCUCAAAAGAGCUUAUAACUUGUAUCCAAACAU
.((((((((((((((..((((((((((((((((((((((((((.(((((((((...))))))))).))))))))))))))))))))))))))..))))))))))))))). (-72.80)
CTGTTTGGATACAAGCCATAAGCT.....................................................................................   total_24nt_0090001_0000004 + 1
...TTTGGATACAAGCCATA.........................................................................................   total_17nt_1980631_0000001 + 4
...TTTGGATACAAGCCATAAGCT.....................................................................................   total_21nt_0187752_0000002 + 4
....TTGGATACAAGCCATAAGCTCT...................................................................................   total_22nt_0338102_0000001 + 5
.........TACAAGCCATAAGCTCTTTTG...............................................................................   total_21nt_0694454_0000001 + 10
.....................................................TAATATATTTTCTGTAGAGAAGCTCT..............................   total_26nt_1696624_0000001 + 54
.......................................................ATATATTTTCTGTAGAGAAGCT................................   total_22nt_0765267_0000005 + 56
.......................................................ATATATTTTCTGTAGAGAAGCTCT..............................   total_24nt_1662578_0000001 + 56
.................................................................TGTAGAGAAGCTCTCAAAAGAGC.....................   total_23nt_0108898_0000001 + 66

gma-MIR4374-N1
>F_18_01_Gm01_-_start_23428261_stop_23428375_length_115nt mappable_miRNA_candidates_14      gma-MIR4374b  94.3   4D17MD89M4D
AGACGGUGCUUACCUCAGCAACCGUCUUUGAAAGUAGGCAUUAUAAGACGUUGCUGAUGUAAGCACCGUCUUUGAAUGCCUACUUUCAAAGUCGGUGUUGAUGUAAGCACCGUCU
(((((((((((((.((((((.(((.(((((((((((((((((..((((((.((((......)))).))))))..))))))))))))))))).))))))))).))))))))))))) (-79.50)
AGACGGTGCTTACCTC...................................................................................................    total_16nt_0821662_0000001 + 1
..................CAACCGTCTTTGAAAG.................................................................................    total_16nt_0437877_0000001 + 19
.....................................................CTGATGTAAGCACCGTCTTTGAAT......................................    total_24nt_1834895_0000001 + 54
.........................................................TGTAAGCACCGTCTTTGAATGCC...................................    total_23nt_0288307_0000001 + 58
.........................................................TGTAAGCACCGTCTTTGAATGCCT..................................    total_24nt_0479263_0000042 + 58
..........................................................GTAAGCACCGTCTTTGAATGCCT..................................    total_23nt_0648578_0000002 + 59
...........................................................TAAGCACCGTCTTTGAATGCCT..................................    total_22nt_0612463_0000010 + 60
............................................................AAGCACCGTCTTTGAATGCCT..................................    total_21nt_0802328_0000004 + 61
.............................................................AGCACCGTCTTTGAATGCCT..................................    total_20nt_0699842_0000002 + 62
.............................................................AGCACCGTCTTTGAATGCCTACTT..............................    total_24nt_2024114_0000001 + 62
.........................................................................GAATGCCTACTTTCAAAGTCGGTG..................    total_24nt_1510247_0000002 + 74
...................................................................................TTTCAAAGTCGGTGTTGATGTAAGC.......    total_25nt_0809991_0000001 + 84
.....................................................................................TCAAAGTCGGTGTTGATGTA..........    total_20nt_1948626_0000001 + 86
.......................................................................................AAAGTCGGTGTTGATGTAAGCACC....    total_24nt_1515550_0000001 + 88

gma-MIR4401-N1
>F_24_01_Gm05_-_start_5369728_stop_5369821_length_94nt mappable_miRNA_candidates_7  gma-MIR4401a  81.9   11I94M11I
GCUUAUUUUCAAAGACGUUGUUGAGGUAAGCACCGUCUUAAGUCUUAAAAUUGUUAAGACGAUGCUUACCUCAACAACGUCUUUGAAAGUAGGC
((((((((((((((((((((((((((((((((.((((((((............)))))))).)))))))))))))))))))))))))))))))) (-62.20)
......TTTCAAAGACGTTGTTGAGGTAAG................................................................    total_24nt_0199750_0000001 + 7
..........AAAGACGTTGTTGAGGTAAGCACC............................................................    total_24nt_0459422_0000006 + 11
.............GACGTTGTTGAGGTAAGCACC............................................................    total_21nt_0900284_0000001 + 14
..............ACGTTGTTGAGGTAAGCACC............................................................    total_20nt_1146133_0000002 + 15
.................................................ATTGTTAAGACGATGCTTACCTCA.....................    total_24nt_0446589_0000001 + 50
......................................................................TCAACAACGTCTTTGAAAGTAGGC    total_24nt_0804229_0000002 + 71
..........................................................................CAACGTCTTTGAAAGT....    total_16nt_0682001_0000002 + 75
```

11 novel gma-MIRs alnout:

```
gma-MIR-N1a
>F_3_01_Gm02_+_start_39925524_stop_39925657_length_134nt mappable_miRNA_candidates_28
GAACUACUUUCUACAUCGGUCGUUUAAUAACCGAUCUAGAAUGUAAUAUUUUCGAUCUAGAAUGUAAUAUUUUCUACAUCGAAAAUAUUACAUUCUAGAUCGGUUAUUAAACGACCAAUGUAGAAAGUAGUUAU
.(((((((((((((((.(((((((((((((((((((((((((((((((((((((((.(((((.........))))).))))))))))))))))))))))))))))))))))).))))))))))))))).. (-87.00)
.......TTTCTACATCGGTCGTTTAATAAC.......................................................................................................      total_24nt_0989988_0000002 + 8
........TTCTACATCGGTCGTTTAATAACC......................................................................................................      total_24nt_0531115_0000002 + 9
..........CTACATCGGTCGTTTAATAAC.......................................................................................................      total_21nt_0759530_0000001 + 11
...........TACATCGGTCGTTTAAT..........................................................................................................      total_17nt_0050829_0000001 + 12
...........TACATCGGTCGTTTAATAAC.......................................................................................................      total_20nt_0842205_0000001 + 12
...........TACATCGGTCGTTTAATAACC......................................................................................................      total_21nt_0368992_0000001 + 12
...........TACATCGGTCGTTTAATAACCG.....................................................................................................      total_22nt_1146662_0000001 + 12
...................TCGTTTAATAACCGATCTAGAATG...........................................................................................      total_24nt_2002156_0000002 + 20
......................TTTAATAACCGATCTAGAATG...........................................................................................      total_21nt_0985664_0000002 + 23
......................TTTAATAACCGATCTAGAATGTAA........................................................................................      total_24nt_0898319_0000001 + 23
.......................TTAATAACCGATCTAGAATG...........................................................................................      total_20nt_0529773_0000001 + 24
.......................TTAATAACCGATCTAGAATGT..........................................................................................      total_21nt_0829385_0000001 + 24
.......................TTAATAACCGATCTAGAATGTAAT.......................................................................................      total_24nt_1527797_0000001 + 24
........................TAATAACCGATCTAGAATGTA.........................................................................................      total_21nt_1398462_0000002 + 25
.........................AATAACCGATCTAGAATGTAAT.......................................................................................      total_22nt_0962388_0000001 + 26
..........................ATAACCGATCTAGAATGTAAT.......................................................................................      total_21nt_0196345_0000010 + 27
..........................ATAACCGATCTAGAATGTAATATT....................................................................................      total_24nt_0951598_0000001 + 27
...........................TAACCGATCTAGAATGTAAT.......................................................................................      total_20nt_1677315_0000002 + 28
.............................ACCGATCTAGAATGTAA........................................................................................      total_17nt_2018820_0000001 + 30
.......................................................................................TTACATTCTAGATCGGTTATT..........................      total_21nt_1555199_0000001 + 88
........................................................................................TACATTCTAGATCGGTTA............................      total_18nt_2086930_0000001 + 89
...........................................................................................ATTCTAGATCGGTTATTAAACGA....................      total_23nt_0461626_0000002 + 92
...........................................................................................ATTCTAGATCGGTTATTAAACGAC...................      total_24nt_0398121_0000003 + 92
............................................................................................TTCTAGATCGGTTATTAAAC......................      total_20nt_0903805_0000001 + 93
............................................................................................TTCTAGATCGGTTATTAAACGA....................      total_22nt_1486811_0000001 + 93
........................................................................................................TATTAAACGACCAATGTAGAAAGT......      total_24nt_1256699_0000001 + 105
..........................................................................................................TTAAACGACCAATGTAGAAAGT......      total_22nt_0310679_0000001 + 107
..............................................................................................................ACGACCAATGTAGAAAGTAGTTAT      total_24nt_1336802_0000001 + 111

gma-MIR-N1b
>F_3_02_Gm02_-_start_39925526_stop_39925654_length_129nt mappable_miRNA_candidates_24
ACUACUUUCUACAUUGGUCGUUUAAUAACCGAUCUAGAAUGUAAUAUUUUCGAUGUAGAAAAUAUUACAUUCUAGAUCGAAAAUAUUACAUUCUAGAUCGGUUAUUAAACGACCGAUGUAGAAAGUAGU
((((((((((((((((((((((((((((((((((((((((((((((((((((((.(((((.........))))).)))))))))))))))))))))))))))))))))))))))))))))))))))))) (-90.90)
.....TTTCTACATTGGTCGTTTAATAAC....................................................................................................    total_24nt_0655528_0000001 + 6
.................TCGTTTAATAACCGATCTAGAATG........................................................................................    total_24nt_2002156_0000002 + 18
....................TTTAATAACCGATCTAGAATG........................................................................................    total_21nt_0985664_0000002 + 21
....................TTTAATAACCGATCTAGAATGTAA.....................................................................................    total_24nt_0898319_0000001 + 21
.....................TTAATAACCGATCTAGAATG........................................................................................    total_20nt_0529773_0000001 + 22
.....................TTAATAACCGATCTAGAATGT.......................................................................................    total_21nt_0829385_0000001 + 22
.....................TTAATAACCGATCTAGAATGTAAT....................................................................................    total_24nt_1527797_0000001 + 22
......................TAATAACCGATCTAGAATGTA......................................................................................    total_21nt_1398462_0000002 + 23
.......................AATAACCGATCTAGAATGTAAT....................................................................................    total_22nt_0962388_0000001 + 24
........................ATAACCGATCTAGAATGTAAT....................................................................................    total_21nt_0196345_0000010 + 25
........................ATAACCGATCTAGAATGTAATATT.................................................................................    total_24nt_0951598_0000001 + 25
.........................TAACCGATCTAGAATGTAAT....................................................................................    total_20nt_1677315_0000002 + 26
...........................ACCGATCTAGAATGTAA.....................................................................................    total_17nt_2018820_0000001 + 28
.....................................................................................TTACATTCTAGATCGGTTATT.......................    total_21nt_1555199_0000001 + 86
......................................................................................TACATTCTAGATCGGTTA.........................    total_18nt_2086930_0000001 + 87
.........................................................................................ATTCTAGATCGGTTATTAAACGA.................    total_23nt_0461626_0000002 + 90
.........................................................................................ATTCTAGATCGGTTATTAAACGAC................    total_24nt_0398121_0000003 + 90
..........................................................................................TTCTAGATCGGTTATTAAAC...................    total_20nt_0903805_0000001 + 91
..........................................................................................TTCTAGATCGGTTATTAAACGA.................    total_22nt_1486811_0000001 + 91
......................................................................................................TATTAAACGACCGATGTAGAAAGT...    total_24nt_1092419_0000013 + 103
.......................................................................................................ATTAAACGACCGATGTAGAAAGT...    total_23nt_0422565_0000001 + 104
........................................................................................................TTAAACGACCGATGTAGAAAGTAG.    total_24nt_0559585_0000001 + 105
.........................................................................................................TAAACGACCGATGTAGAAAGTAGT    total_24nt_0238945_0000001 + 106
...............................................................................................................ACCGATGTAGAAAGT...    total_15nt_0378749_0000002 + 112
```

gma-MIR-N1c

```
>F_3_03_Gm03_+_start_11919586_stop_11919706_length_121nt mappable_miRNA_candidates_15
AAAACUACUUUCUACAUCGGUCGUUUAAUAACGGAUCUAGAAUGUAAUUUUUUUCUACGUUGUUGUAGAAAAUAUUACAUUCUAGAUCGGUUAUUAAACGACCGAUGUAGAAAGUAGUUAU
..((((((((((((((((((((((((((((((.(((((((((((((((..(((((((((....))))))))).))))))))))))))).)))))))))))))))))))))))))))))).. (-80.20)
........TTTCTACATCGGTCGTTTAATAAC.........................................................................................     total_24nt_0989988_0000002 + 9
...........CTACATCGGTCGTTTAATAAC.........................................................................................     total_21nt_0759530_0000001 + 12
............TACATCGGTCGTTTAAT............................................................................................     total_17nt_0050829_0000001 + 13
............TACATCGGTCGTTTAATAAC.........................................................................................     total_20nt_0842205_0000001 + 13
..........................................................................TTACATTCTAGATCGGTTATT..........................     total_21nt_1555199_0000001 + 75
...........................................................................TACATTCTAGATCGGTTA............................     total_18nt_2086930_0000001 + 76
..............................................................................ATTCTAGATCGGTTATTAAACGA....................     total_23nt_0461626_0000002 + 79
..............................................................................ATTCTAGATCGGTTATTAAACGAC...................     total_24nt_0398121_0000003 + 79
...............................................................................TTCTAGATCGGTTATTAAAC......................     total_20nt_0903805_0000001 + 80
...............................................................................TTCTAGATCGGTTATTAAACGA....................     total_22nt_1486811_0000001 + 80
...........................................................................................TATTAAACGACCGATGTAGAAAGT......     total_24nt_1092419_0000013 + 92
............................................................................................ATTAAACGACCGATGTAGAAAGT......     total_23nt_0422565_0000001 + 93
.............................................................................................TTAAACGACCGATGTAGAAAGTAG....     total_24nt_0559585_0000001 + 94
..............................................................................................TAAACGACCGATGTAGAAAGTAGT...     total_24nt_0238945_0000001 + 95
....................................................................................................ACCGATGTAGAAAGT......     total_15nt_0378749_0000002 + 101
```

gma-MIR-N1d

```
>F_3_04_Gm03_-_start_11919589_stop_11919703_length_115nt mappable_miRNA_candidates_24
ACUACUUUCUACAUCGGUCGUUUAAUAACCGAUCUAGAAUGUAAUAUUUUCUACAACAACGUAGAAAAAAAUUACAUUCUAGAUCCGUUAUUAAACGACCGAUGUAGAAAGUAGU
(((((((((((((((((((((((((((((.(((((((((((((((.((((((((......))))))))..))))))))))))))).))))))))))))))))))))))))))))) (-75.20)
.....TTTCTACATCGGTCGTTTAATAAC......................................................................................    total_24nt_0989988_0000002 + 6
......TTCTACATCGGTCGTTTAATAACC.....................................................................................    total_24nt_0531115_0000002 + 7
........CTACATCGGTCGTTTAATAAC......................................................................................    total_21nt_0759530_0000001 + 9
.........TACATCGGTCGTTTAAT.........................................................................................    total_17nt_0050829_0000001 + 10
.........TACATCGGTCGTTTAATAAC......................................................................................    total_20nt_0842205_0000001 + 10
.........TACATCGGTCGTTTAATAACC.....................................................................................    total_21nt_0368992_0000001 + 10
.........TACATCGGTCGTTTAATAACCG....................................................................................    total_22nt_1146662_0000001 + 10
.................TCGTTTAATAACCGATCTAGAATG..........................................................................    total_24nt_2002156_0000002 + 18
....................TTTAATAACCGATCTAGAATG..........................................................................    total_21nt_0985664_0000002 + 21
....................TTTAATAACCGATCTAGAATGTAA.......................................................................    total_24nt_0898319_0000001 + 21
.....................TTAATAACCGATCTAGAATG..........................................................................    total_20nt_0529773_0000001 + 22
.....................TTAATAACCGATCTAGAATGT.........................................................................    total_21nt_0829385_0000001 + 22
.....................TTAATAACCGATCTAGAATGTAAT......................................................................    total_24nt_1527797_0000001 + 22
......................TAATAACCGATCTAGAATGTA........................................................................    total_21nt_1398462_0000002 + 23
.......................AATAACCGATCTAGAATGTAAT......................................................................    total_22nt_0962388_0000001 + 24
........................ATAACCGATCTAGAATGTAAT......................................................................    total_21nt_0196345_0000010 + 25
........................ATAACCGATCTAGAATGTAATATT...................................................................    total_24nt_0951598_0000001 + 25
.........................TAACCGATCTAGAATGTAAT......................................................................    total_20nt_1677315_0000002 + 26
...........................ACCGATCTAGAATGTAA.......................................................................    total_17nt_2018820_0000001 + 28
........................................................................................TATTAAACGACCGATGTAGAAAGT...    total_24nt_1092419_0000013 + 89
.........................................................................................ATTAAACGACCGATGTAGAAAGT...    total_23nt_0422565_0000001 + 90
..........................................................................................TTAAACGACCGATGTAGAAAGTAG.    total_24nt_0559585_0000001 + 91
...........................................................................................TAAACGACCGATGTAGAAAGTAGT    total_24nt_0238945_0000001 + 92
.................................................................................................ACCGATGTAGAAAGT...    total_15nt_0378749_0000002 + 98
```

```
gma-MIR-N2
>F_12_01_Gm06_-_start_46035186_stop_46035339_length_154nt mappable_miRNA_candidates_49
UCUUACUGCUACAGUACUAUUCAACUAAAAUAUUAGACUGCUGUACUUCCAGGAAGUACUGUUCAAACAUUUGUUGUACAGUAAAUGUUUGAACAGUACUUCCUGGAAGUAUAGCAGUCUAAUAUUUUAGUUGAAUGGUAUUGUAGUACUAAGA
(((((.(((((((((((((((((((((((((((((((((((((((((((((((((((((((((((((((((((........))))))))))))))))))))))))))))))))))))))))))))))))))))))))))))))))))).))))) (-111.50)
................CTATTCAACTAAAATATTAGAC....................................................................................................................	total_22nt_0780151_0000001 + 17
.........................TAAAATATTAGACTGCTGT..............................................................................................................	total_19nt_1058460_0000001 + 26
.........................TAAAATATTAGACTGCTGTACT...........................................................................................................	total_22nt_0939589_0000068 + 26
.........................TAAAATATTAGACTGCTGTACTT..........................................................................................................	total_23nt_1879373_0000001 + 26
..........................AAAATATTAGACTGCTGTACT...........................................................................................................	total_21nt_1717864_0000002 + 27
...........................AAATATTAGACTGCTGTACT...........................................................................................................	total_20nt_0392158_0000004 + 28
...........................AAATATTAGACTGCTGTACTTCC........................................................................................................	total_23nt_0549433_0000001 + 28
............................AATATTAGACTGCTGTACT...........................................................................................................	total_19nt_1082852_0000001 + 29
............................AATATTAGACTGCTGTACTTC.........................................................................................................	total_21nt_0499859_0000001 + 29
.............................ATATTAGACTGCTGTACT...........................................................................................................	total_18nt_1124454_0000001 + 30
.............................ATATTAGACTGCTGTACTTCC........................................................................................................	total_21nt_1421401_0000001 + 30
..............................TATTAGACTGCTGTACTTCC........................................................................................................	total_20nt_1299850_0000001 + 31
..............................TATTAGACTGCTGTACTTCCAG......................................................................................................	total_22nt_0911681_0000006 + 31
...............................ATTAGACTGCTGTACTTCCAG......................................................................................................	total_21nt_0254748_0000001 + 32
................................TTAGACTGCTGTACTTCC........................................................................................................	total_18nt_2025910_0000001 + 33
................................TTAGACTGCTGTACTTCCAG......................................................................................................	total_20nt_0458466_0000002 + 33
.................................TAGACTGCTGTACTTCCAG......................................................................................................	total_19nt_1698647_0000001 + 34
..................................AGACTGCTGTACTTCC........................................................................................................	total_16nt_1713642_0000001 + 35
..............................................TTCCAGGAAGTACTGTTCAAAC......................................................................................	total_22nt_1252927_0000001 + 47
.................................................................................TAAATGTTTGAACAGTACTTCC...................................................	total_22nt_1655054_0000001 + 82
..................................................................................AAATGTTTGAACAGTACTTCCT..................................................	total_22nt_1281020_0000001 + 83
.....................................................................................TGTTTGAACAGTACTTCCT..................................................	total_19nt_1706507_0000002 + 86
.....................................................................................TGTTTGAACAGTACTTCCTG.................................................	total_20nt_0053218_0000001 + 86
.....................................................................................TGTTTGAACAGTACTTCCTGGA...............................................	total_22nt_2000437_0000002 + 86
......................................................................................GTTTGAACAGTACTTCC...................................................	total_17nt_1751079_0000001 + 87
.......................................................................................TTTGAACAGTACTTCC...................................................	total_16nt_2066322_0000002 + 88
.......................................................................................TTTGAACAGTACTTCCTG.................................................	total_18nt_1794522_0000001 + 88
.......................................................................................TTTGAACAGTACTTCCTGGA...............................................	total_20nt_1973323_0000002 + 88
........................................................................................TTGAACAGTACTTCCTGGA...............................................	total_19nt_0036152_0000001 + 89
........................................................................................TTGAACAGTACTTCCTGGAAG.............................................	total_21nt_1376476_0000001 + 89
........................................................................................TTGAACAGTACTTCCTGGAAGTA...........................................	total_23nt_1961480_0000001 + 89
.........................................................................................TGAACAGTACTTCCTGGAAGT............................................	total_21nt_1513692_0000001 + 90
.......................................................................................................................TAATATTTTAGTTGAATGGT...............	total_20nt_0742429_0000001 + 120
.......................................................................................................................TAATATTTTAGTTGAATGGTA..............	total_21nt_0951677_0000002 + 120
.........................................................................................................................ATATTTTAGTTGAATGGTATT............	total_21nt_0259632_0000001 + 122
..........................................................................................................................TATTTTAGTTGAATGGTATTG...........	total_21nt_1602892_0000001 + 123
............................................................................................................................TTTTAGTTGAATGGTATTGTAGT.......	total_23nt_0414884_0000001 + 125
.............................................................................................................................TTTAGTTGAATGGTATTGTAGT.......	total_22nt_1463594_0000002 + 126
.............................................................................................................................TTTAGTTGAATGGTATTGTAGTACT....	total_25nt_1016853_0000001 + 126
..............................................................................................................................TTAGTTGAATGGTATTGTAGTA......	total_22nt_0619312_0000008 + 127
..............................................................................................................................TTAGTTGAATGGTATTGTAGTACT....	total_24nt_0621070_0000013 + 127
...............................................................................................................................TAGTTGAATGGTATTGTAGT.......	total_20nt_0138271_0000001 + 128
...............................................................................................................................TAGTTGAATGGTATTGTAGTACT....	total_23nt_1835226_0000003 + 128
................................................................................................................................AGTTGAATGGTATTGTAGTACT....	total_22nt_1181299_0000001 + 129
.................................................................................................................................GTTGAATGGTATTGTAGTACT....	total_21nt_1447359_0000001 + 130
..................................................................................................................................TTGAATGGTATTGTAGTAC.....	total_19nt_1273336_0000001 + 131
..................................................................................................................................TTGAATGGTATTGTAGTACT....	total_20nt_1916479_0000002 + 131
..................................................................................................................................TTGAATGGTATTGTAGTACTA...	total_21nt_0806339_0000001 + 131
...................................................................................................................................TGAATGGTATTGTAGTACT....	total_19nt_0390860_0000002 + 132
```

```
gma-MIR-N3
>F_16_01_Gm07_-_start_19042117_stop_19042232_length_116nt mappable_miRNA_candidates_6      gma-MIR5377   66.4   2I17MI18MD36M2I44M2I
CCGAUGUUGAAUUUUGACAUUCUACAUCGGUUAUUAGGACAACCGAUGUAGAAUGACCAUCAUUCUACAUCGGGUAUGUACUGAACCGAUGUAGAAUGCUAGACAUUCAACAUCGG
((((((((((((((.((((((((((((((..((((.(((.((((((((((((((...))))))))))))))...))).)))))))))))))))))).)))).)))))))))))) (-65.90)
.................CATTCTACATCGGTTATTA................................................................................   total_19nt_0112577_0000001 + 18
..................ATTCTACATCGGTTATTAGGA.............................................................................   total_21nt_0721261_0000001 + 19
.....................CTACATCGGTTATTAGGA.............................................................................   total_18nt_0495544_0000001 + 22
................................................................................CTGAACCGATGTAGAAT...................   total_17nt_0321846_0000001 + 81
...................................................................................AACCGATGTAGAATGCTAGACA...........   total_22nt_1260063_0000003 + 84
...................................................................................AACCGATGTAGAATGCTAGACATT.........   total_24nt_0223862_0000025 + 84


gma-MIR-N4
>F_10_01_Gm02_-_start_48422489_stop_48422679_length_191nt mappable_miRNA_candidates_24
AUCAAUAAAAGAAGAAUAAAAAUUAAGUGCACAGAUUGAGUAGAUGUUUUAUCUACCCGAAGUCCAAAGACAAGAAAUUAUCUACCAGCACUGAUUGAUCAUUGCUGGUGGAUAAUUUCUUGACUUUGGACUUUUGGGUACACAAAGCACCUACUCAAUCUAUUCACUUAAUUUGUGUUCUUUUCAUUGAC
.((((...((((((((.((((((((((...((((((((((.((((((...(((((((((((((((.((((((((((((((((((((.(((....))).)))))))))))))))))))))).))))))))).)))))))))...)))))).)))))))))))...))))))))))).)))))))))).))))). (-99.20)
...............................CAGATTGAGTAGATGTTTTATC..........................................................................................................................................    total_22nt_1432163_0000002 + 32
...................................................TCTACCCGAAGTCCAAAGACAA......................................................................................................................    total_22nt_1081861_0000001 + 52
........................................................CCGAAGTCCAAAGACAAGA....................................................................................................................    total_19nt_0052196_0000001 + 57
........................................................CCGAAGTCCAAAGACAAGAAAT.................................................................................................................    total_22nt_1210077_0000003 + 57
..............................................................................TATCTACCAGCACAA..................................................................................................    total_15nt_0386967_0000001 + 79
........................................................................................................CTGGTGGATAATTTCTTGACT..................................................................    total_21nt_0073789_0000003 + 105
................................................................................................................TAATTTCTTGACTTTGGACT...........................................................    total_20nt_0348375_0000002 + 113
................................................................................................................TAATTTCTTGACTTTGGACTT..........................................................    total_21nt_1936805_0000001 + 113
.................................................................................................................AATTTCTTGACTTTGGACTTT.........................................................    total_21nt_1408709_0000001 + 114
..................................................................................................................ATTTCTTGACTTTGGACTTTT........................................................    total_21nt_2101258_0000003 + 115
..................................................................................................................ATTTCTTGACTTTGGACTTTTGGGT....................................................    total_25nt_1827936_0000001 + 115
...................................................................................................................TTTCTTGACTTTGGAC............................................................    total_16nt_1813855_0000001 + 116
...................................................................................................................TTTCTTGACTTTGGACTTTTG.......................................................    total_21nt_1791939_0000003 + 116
...................................................................................................................TTTCTTGACTTTGGACTTTTGGG.....................................................    total_23nt_1514436_0000001 + 116
....................................................................................................................TTCTTGACTTTGGACTTTTGGG.....................................................    total_22nt_1415522_0000001 + 117
.....................................................................................................................TCTTGACTTTGGACTTT.........................................................    total_17nt_0096092_0000001 + 118
.....................................................................................................................TCTTGACTTTGGACTTTTGG......................................................    total_20nt_0835538_0000001 + 118
.....................................................................................................................TCTTGACTTTGGACTTTTGGG.....................................................    total_21nt_0570123_0000005 + 118
.....................................................................................................................TCTTGACTTTGGACTTTTGGGT....................................................    total_22nt_0629199_0000020 + 118
.....................................................................................................................TCTTGACTTTGGACTTTTGGGTA...................................................    total_23nt_0112674_0000004 + 118
......................................................................................................................CTTGACTTTGGACTTTTGGGTA...................................................    total_22nt_0094989_0000001 + 119
.......................................................................................................................TTGACTTTGGACTTTTGGGTACA.................................................    total_23nt_0201906_0000001 + 120
.......................................................................................................................TTGACTTTGGACTTTTGGGTACACA...............................................    total_25nt_1984761_0000001 + 120
........................................................................................................................TGACTTTGGACTTTTGGGT....................................................    total_19nt_1930618_0000001 + 121

gma-MIR-N5
>F_29_01_Gm07_+_start_44428050_stop_44428136_length_87nt mappable_miRNA_candidates_14      mtr-MIR2676e  62.1   246I87M18I
UAUGUUUGGAUAGAGAAUUUUAAAUAAGGAAAAUAAUUUACCAGAGAAUUUGAAUUUUUGUAAUUUAGAAUUUAUUGUUUGGAUGUU
.(((((((((((.((((((((((.(.((((((((((......)))))...)))))).).))))))))))).))))))))))))). (-16.50)
TATGTTTGGATAGAGAA......................................................................    total_17nt_0333444_0000001 + 1
TATGTTTGGATAGAGAATT....................................................................    total_19nt_0400021_0000001 + 1
TATGTTTGGATAGAGAATTTTAA................................................................    total_23nt_0928588_0000007 + 1
TATGTTTGGATAGAGAATTTTAAA...............................................................    total_24nt_1100601_0000011 + 1
.ATGTTTGGATAGAGAATTTTAA................................................................    total_22nt_0921662_0000001 + 2
.ATGTTTGGATAGAGAATTTTAAA...............................................................    total_23nt_0347489_0000002 + 2
.ATGTTTGGATAGAGAATTTTAAAT..............................................................    total_24nt_0901266_0000001 + 2
..TGTTTGGATAGAGAATTTTAA................................................................    total_21nt_0600375_0000003 + 3
....TTTGGATAGAGAATTTTAAA...............................................................    total_20nt_1238433_0000001 + 5
....TTTGGATAGAGAATTTTAAAT..............................................................    total_21nt_1903931_0000001 + 5
.....TTGGATAGAGAATTTTAAA...............................................................    total_19nt_0121330_0000001 + 6
............................................................TAATTTAGAATTTATTGTTTGGA....    total_23nt_0830533_0000001 + 61
.............................................................AATTTAGAATTTATTGTTTGGATG..    total_24nt_0086439_0000001 + 62
...............................................................TTTAGAATTTATTGTTTGGATGTT    total_24nt_0009906_0000001 + 64
```

```
gma-MIR-N6
>F_11_01_Gm05_+_start_40112942_stop_40113095_length_154nt mappable_miRNA_candidates_39
UUUUAGGAGUAUGCUAGCAAUAUACCACUUUGGAUUUGAUAUACUUCUAAAGGUGUAACUUGUUUAAUCUAAUAAUUUUUAGGAGUAUAUUAAACGAGUUACACCUCUAGAAGUAGAUCAAAUCCAAAGUUGUAUAUUGCUAACAUACUCCUAA
..((((((((((.((((((((((.(((((((((((((.(((((((.((((((((((((((((((((((((((......))))......))))))))))))))))))).)))))))).)))))))))))))).))))))))))).)))))))))) (-87.40)
TTTTAGGAGTATGCTAGCAATA....................................................................................................................................       total_22nt_0026999_0000001 + 1
..TTAGGAGTATGCTAGCAATATA..................................................................................................................................       total_22nt_0631059_0000001 + 3
..TTAGGAGTATGCTAGCAATATACC................................................................................................................................       total_24nt_0924988_0000002 + 3
.........TATGCTAGCAATATACCACTT............................................................................................................................       total_21nt_0598440_0000002 + 10
...........TGCTAGCAATATACCACTTTGGAT.......................................................................................................................       total_24nt_2104754_0000003 + 12
.............CTAGCAATATACCACTTTGGAT.......................................................................................................................       total_22nt_1251627_0000001 + 14
..............TAGCAATATACCACTTTGGAT.......................................................................................................................       total_21nt_0649097_0000005 + 15
..............TAGCAATATACCACTTTGGATTT.....................................................................................................................       total_23nt_0554526_0000001 + 15
................GCAATATACCACTTTGGATTT.....................................................................................................................       total_21nt_0458927_0000001 + 17
.....................ATACCACTTTGGATTTGATATACT.............................................................................................................       total_24nt_0041352_0000001 + 22
......................TACCACTTTGGATTTGATATA...............................................................................................................       total_21nt_1501665_0000001 + 23
........................CCACTTTGGATTTGATA.................................................................................................................       total_17nt_1429409_0000001 + 25
.........................CACTTTGGATTTGATATA...............................................................................................................       total_18nt_1409776_0000001 + 26
.........................CACTTTGGATTTGATATACT.............................................................................................................       total_20nt_0344224_0000001 + 26
.........................CACTTTGGATTTGATATACTT............................................................................................................       total_21nt_1066537_0000006 + 26
..........................ACTTTGGATTTGATATACT.............................................................................................................       total_19nt_0542056_0000001 + 27
..........................ACTTTGGATTTGATATACTTCT..........................................................................................................       total_22nt_1758304_0000002 + 27
...........................CTTTGGATTTGATATACT.............................................................................................................       total_18nt_1986557_0000001 + 28
...........................CTTTGGATTTGATATACTTCT..........................................................................................................       total_21nt_0423992_0000001 + 28
...........................CTTTGGATTTGATATACTTCTA.........................................................................................................       total_22nt_0039230_0000004 + 28
............................TTTGGATTTGATATACTTCTA.........................................................................................................       total_21nt_1586117_0000001 + 29
.............................TTGGATTTGATATACTTCTA.........................................................................................................       total_20nt_0457101_0000003 + 30
.............................TTGGATTTGATATACTTCTAA........................................................................................................       total_21nt_0431137_0000001 + 30
.................................ATTTGATATACTTCTAAAGGTGT..................................................................................................       total_23nt_0278411_0000001 + 34
..................................TTTGATATACTTCTAAAGGTG...................................................................................................       total_21nt_1696786_0000002 + 35
....................................................................................................ACACCTCTAGAAGTAGA.....................................       total_17nt_1821831_0000001 + 101
.....................................................................................................CACCTCTAGAAGTAGATCAAAT...............................       total_22nt_0918862_0000001 + 102
.........................................................................................................TCTAGAAGTAGATCAAATCC.............................       total_20nt_1402283_0000001 + 106
..........................................................................................................CTAGAAGTAGATCAAATCCA............................       total_20nt_1692874_0000001 + 107
..........................................................................................................CTAGAAGTAGATCAAATCCAA...........................       total_21nt_1559228_0000001 + 107
..........................................................................................................CTAGAAGTAGATCAAATCCAAA..........................       total_22nt_1482441_0000001 + 107
...........................................................................................................TAGAAGTAGATCAAATCCAAA..........................       total_21nt_0370200_0000001 + 108
...........................................................................................................TAGAAGTAGATCAAATCCAAAGTT.......................       total_24nt_0001048_0000001 + 108
.............................................................................................................GAAGTAGATCAAATCCAAAGTT.......................       total_22nt_0134770_0000003 + 110
..............................................................................................................AAGTAGATCAAATCCAAAGTT.......................       total_21nt_1204198_0000002 + 111
.....................................................................................................................TCAAATCCAAAGTTGTATATTG...............       total_22nt_1680000_0000002 + 118
......................................................................................................................CAAATCCAAAGTTGTATATTGCT.............       total_23nt_0038618_0000001 + 119
.................................................................................................................................TTGTATATTGCTAACATACTC....       total_21nt_0779488_0000001 + 130
.................................................................................................................................TTGTATATTGCTAACATACTCCT..       total_23nt_0005689_0000001 + 130

gma-MIR-N7
>F_13_01_Gm07_+_start_1788665_stop_1788817_length_153nt mappable_miRNA_candidates_7 der-mir-281-2 64.1   8D10M6D20MI8M3D23M9D8M2D22M34D
AACCUAAUGGAAUUGUGAUCCCGUUAUGUAAUAAUUUAUCUAUCACACUGAAAUCGUGAUUUCAUUGUGUAAUAAUUUGUAUAACACAAUGGAAUCGUGAUUUCGUUGUGUUAGACAAAUUAUUAUACAACGGAAUCAUGGUUCUAUUGUGUU
.((.(((((((((((((.(((((.(((((((((((.((((.((((.(((((((((((((((((((((((.(((.....))).))))))))))))))))))))))).)))).)))).))))))))))).))))).))))))))))))).)). (-69.20)
....................................................ATCGTGATTTCATTGTGT................................................................................... total_18nt_1560360_0000001 + 53
..................................................................................AACACAATGGAATCGTGATTTCGT............................................... total_24nt_0244453_0000005 + 83
.............................................................................................ATCGTGATTTCGTTGTGTTAGACA.................................... total_24nt_1392523_0000001 + 94
..............................................................................................TCGTGATTTCGTTGTGTTAGA...................................... total_21nt_2026292_0000001 + 95
.................................................................................................TGATTTCGTTGTGTTAGACAA................................... total_21nt_1204086_0000001 + 98
......................................................................................................................ATTATTATACAACGGAATCATGGT........... total_24nt_1769771_0000001 + 119
.................................................................................................................................ACGGAATCATGGTTCTATTGTGTT total_24nt_0393725_0000001 + 130

gma-MIR-N8
>F_34_01_Gm08_-_start_4271026_stop_4271087_length_62nt mappable_miRNA_candidates_5
AUUUAGUUUUGAUAUCACUGUCCAAAGAAAAAUAUCUUGGGACAGAGGUAUCAAUACUAAAU
(((((((.((((((((.((((((.((((......)))).)))))).)))))))).))))))) (-27.50)
..TTAGTTTTGATATCACTGTCC....................................... total_21nt_0120496_0000001 + 3
..TTAGTTTTGATATCACTGTCCAAAG................................... total_25nt_0784113_0000001 + 3
...TAGTTTTGATATCACTGTC........................................ total_19nt_1270975_0000001 + 4
...TAGTTTTGATATCACTGTCCAAAG................................... total_24nt_1397672_0000005 + 4
.....................................TGGGACAGAGGTATCAATACTAAAT total_25nt_0282595_0000003 + 38
```
