# Supplementary material for: Comparative Profiling of microRNA Expression in Soybean Seeds from Genetically Modified Plants and their Near-Isogenic Parental Lines
Source: PLoS One. 2016 May 23;11(5):e0155896. doi: 10.1371/journal.pone.0155896 (PMC4876996; doi:10.1371/journal.pone.0155896)
Supplement: S1 File — (DOCX) [file pone.0155896.s005.docx]

**Sanger_sequencing_results_of_novel_miRNAs_20160405**

1.gma-MIR1516-N1

ATATATTTTCTGTAGAGAAGCT

>1-12-PJET1-2-forward_B01.ab1

ATCAACTTCGATGCTCGAGTTTTTCAGCAAGATGCTGTCAACGATACGCTACGTAACGGCATGACAGTGTTTTTTTTTTTTTTTTTTTTTAAAATACTAGCTTCTCTACAGAAAATATATGATCTTTCTAGAAGATCTCCTACAATATTCTCAGCTGCCATGGAAAATCGATGTTCTTCTTTTATTCTCTCAAGATTTTCAGGCTGTATATTAAAACTTATATTAAGAACTATGCTAACCACCTCATCAGGAACCGTTGTAGGTGGCGTGGGTTTTCTTGGCAATCGACTCTCATGAAAACTACGAGCTAAATATTCAATATGTTCCTCTTGACCAACTTTATTCTGCATTTTTTTTGAACGAGGTTTAGAGCAAGCTTCAGGAAACTGAGACAGGAATTTTATTAAAAATTTAAATTTTGAAGAAAGTTCAGGGTTAATAGCATCCATTTTTTGCTTTGCAAGTTCCTCAGCATTCTTAACAAAAGACGTCTCTTTTGACATGTTTAAAGTTTAAACCTCCTGTGTGAAATTATTATCCGCTCATAATTCCACACATTATACGAGCCGGAAGCATAAAGTGTAAAGCCTGGGGTGCCTAATGAGTGAGCTAACTCACATTAATTGCGTTGCGCTCACTGCCAATTGCTTTCCAGTCGGGAAACCTGTCGTGCCAGCTGCATTAATGAATCGGCCAACGCGCGGGGAGAGGCGGTTTGCGTATTGGGCGCTCTTCCGCTTCCTCGCTCACTGACTCGCTGCGCTCGGTCGTTCGGCTGCGGCGAGCGGTATCAGCTCACTCAAAGGCGGTAATACGGTTATCCACAGAATCAGGGGATAACGCAGAAAGAACATGTGAGCAAAAGGCCAGCAAAAGGCCAGGAACCGTAAAAAGGCCGCGTTGCTGGCGTTTTTCCATAGGCTCCGCCCCCCTGACGAGCATCACAAAAATCGACGCTCAAGTCAGAGTGGCGAAACCCGACAGGACTATAAAGATACCAGCGTTTCCCCCTGTAGCTCCTCGTGCGCTCTCTGTCGACCCTGCGCTACGGAATACTGTCGCTTCTCCTCGGAGCGTGGCGCTTTCCTCATAAGCTCACGCTGTAGTATCTCAGTCGGTGTAAGGTCGTCGCCCAGCTGGCTGGTGTGACCGACCCCGGTTAGCGACGCGTGGCCTACCGGTACATCGCTGAGTCACCGTGAACGACTTTTCCCCCTGGGGC

2. gma-MIR4374-N1

TGTAAGCACCGTCTTTGAATGCC

>2-10-PJET1-2-forward_H01.ab1

CCCCCACTTCCGGATGGCTCGAGTTTTTCAGCAGATGTAAGCACCGTCTTTGAATGCCAAAAAAAAAAAAAAAAAAAAACACTGTCATGCCGTTACGTAGCGTATCGTTGACAGCATCTTTCTAGAAGATCTCCTACAATATTCTCAGCTGCCATGGAAAATCGATGTTCTTCTTTTATTCTCTCAAGATTTTCAGGCTGTATATTAAAACTTATATTAAGAACTATGCTAACCACCTCATCAGGAACCGTTGTAGGTGGCGTGGGTTTTCTTGGCAATCGACTCTCATGAAAACTACGAGCTAAATATTCAATATGTTCCTCTTGACCAACTTTATTCTGCATTTTTTTTGAACGAGGTTTAGAGCAAGCTTCAGGAAACTGAGACAGGAATTTTATTAAAAATTTAAATTTTGAAGAAAGTTCAGGGTTAATAGCATCCATTTTTTGCTTTGCAAGTTCCTCAGCATTCTTAACAAAAGACGTCTCTTTTGACATGTTTAAAGTTTAAACCTCCTGTGTGAAATTATTATCCGCTCATAATTCCACACATTATACGAGCCGGAAGCATAAAGTGTAAAGCCTGGGGTGCCTAATGAGTGAGCTAACTCACATTAATTGCGTTGCGCTCACTGCCAATTGCTTTCCAGTCGGGAAACCTGTCGTGCCAGCTGCATTAATGAATCGGCCAACGCGCGGGGAGAGGCGGTTTGCGTATTGGGCGCTCTTCCGCTTCCTCGCTCACTGACTCGCTGCGCTCGGTCGTTCGGCTGCGGCGAGCGGTATCAGCTCACTCAAAGGCGGTAATACGGTTATCCACAGAATCAGGGGATAACGCACGAAAGAACATGTGAGCAAAAGGCCAGCAAAAGGCCAGGAACCGTAAAAAGGCCGCGTTGCTGGCGTTTTTCCATAGGCTCCGCCCCCCTGACGAGCATCACAAAAATCGACGCTCAAGTCAGAGGTGGCGAAACCCGACAGGACTATAAAGATACCAGGCGTTTCCCCCTGTAGCTCCTCGTGCGCTCTCTGTTCGACCTGCGCTACGATACTGTCGCTTTCTCCTTCGGGAGCGTGCGCTTTCTCATAGCTCACGCTGTAAGTATCTCAGTCGGTGTAGTCGTCGCCTCAAGCCTGGCCTGTGTGCACGACCCCGTTCAGCGACGCTTGGACTTATTCGGTAACATTGCTTGGATCAACA

GTTGAAACGACTATTGTCACTGGGACAGACACACCTGGGTA

3. gma-MIR4401-N1

AGACGTTGTTGAGGTAAGCACC

>3-9-PJET1-2-forward_H01.ab1

TTTCCCTAATTTCGGATGCTCGAGTTTTTCAGCAGATGCTGTCAACGATACGCTACGTAACGGCATGACAGTGTTTTTTTTTTTTTTTTTTTTTTTGGTGCTTACCTCAACAACGTCTATCTTTCTAGAAGATCTCCTACAATATTCTCAGCTGCCATGGAAAATCGATGTTCTTCTTTTATTCTCTCAAGATTTTCAGGCTGTATATTAAAACTTATATTAAGAACTATGCTAACCACCTCATCAGGAACCGTTGTAGGTGGCGTGGGTTTTCTTGGCAATCGACTCTCATGAAAACTACGAGCTAAATATTCAATATGTTCCTCTTGACCAACTTTATTCTGCATTTTTTTTGAACGAGGTTTAGAGCAAGCTTCAGGAAACTGAGACAGGAATTTTATTAAAAATTTAAATTTTGAAGAAAGTTCAGGGTTAATAGCATCCATTTTTTGCTTTGCAAGTTCCTCAGCATTCTTAACAAAAGACGTCTCTTTTGACATGTTTAAAGTTTAAACCTCCTGTGTGAAATTATTATCCGCTCATAATTCCACACATTATACGAGCCGGAAGCATAAAGTGTAAAGCCTGGGGTGCCTAATGAGTGAGCTAACTCACATTAATTGCGTTGCGCTCACTGCCAATTGCTTTCCAGTCGGGAAACCTGTCGTGCCAGCTGCATTAATGAATCGGCCAACGCGCGGGGAGAGGCGGTTTGCGTATTGGGCGCTCTTCCGCTTCCTCGCTCACTGACTCGCTGCGCTCGGTCGTTCGGCTGCGGCGAGCGGTATCAGCTCACTCAAAGGCGGTAATACGGTTATCCACAGAATCAGGGGATAACGCAGAAAGAACATGTGAGCAAAAGCCAGCAAAAAGCCAGAACCGTAAAAAGGCCGCGTTGCTGGCGTTTTTCCATAGGCTCCGCCCCCCTGACGAGCATCACAAAAATCGACGCTCAAGTCAGAGTGGCGAAACCCGACAGGACTATAAAGATACCAGCGTTTCCCCTGGAAGCTCCCTCGTGCGCTCTCCTGTCCGACCTGCGCTACCGGATACCTGTCGCTTTCTCCCTCGGGAGCGTGCGCTTTCTCATAGCTCACGCTGTAGTATCTCAGTCGGTGTAGTCGGTCGCTTCAGCTGGGCTGGTGTGCACGAACCCCCGGTTCAGCGGAACGCGTGGCACTATCGGTACTATTCGTCTGGATCCACCGGTGAGACACGCCACTAATCGCCCACAACT

4. gma-MIR-N1a

ATAACCGATCTAGAATGTAAT

>4-9-PJET1-2-forward_D02.ab1

ATCCTAACTCCCGATGGCTCGAGTTTTTAGCAGATTCAACGATACGCTACGTAACGGCATGACAGTGTTTTTTTTTTTTTTTTTTTTTTGAGACCATTACATTCTAGATCGGTTATGCATCTTTCTAGAAGATCTCCTACAATATTCTCAGCTGCCATGGAAAATCGATGTTCTTCTTTTATTCTCTCAAGATTTTCAGGCTGTATATTAAAACTTATATTAAGAACTATGCTAACCACCTCATCAGGAACCGTTGTAGGTGGCGTGGGTTTTCTTGGCAATCGACTCTCATGAAAACTACGAGCTAAATATTCAATATGTTCCTCTTGACCAACTTTATTCTGCATTTTTTTTGAACGAGGTTTAGAGCAAGCTTCAGGAAACTGAGACAGGAATTTTATTAAAAATTTAAATTTTGAAGAAAGTTCAGGGTTAATAGCATCCATTTTTTGCTTTGCAAGTTCCTCAGCATTCTTAACAAAAGACGTCTCTTTTGACATGTTTAAAGTTTAAACCTCCTGTGTGAAATTATTATCCGCTCATAATTCCACACATTATACGAGCCGGAAGCATAAAGTGTAAAGCCTGGGGTGCCTAATGAGTGAGCTAACTCACATTAATTGCGTTGCGCTCACTGCCAATTGCTTTCCAGTCGGGAAACCTGTCGTGCCAGCTGCATTAATGAATCGGCCAACGCGCGGGGAGAGGCGGTTTGCGTATTGGGCGCTCTTCCGCTTCCTCGCTCACTGACTCGCTGCGCTCGGTCGTTCGGCTGCGGCGAGCGGTATCAGCTCACTCAAAGGCGGTAATACGGTTATCCACAGAATCAGGGGATAACGCAGGAAAGAACATGTGAGCAAAAGGCCAGCAAAAGGCCAGGAACCGTAAAAAGGCCGCGTTGCTGGCGTTTTTCCATAGGCTCCGCCCCCCTGACGAGCATCACAAAAATCGACGCTCAAGTCAGAGTGGCGAAACCCGACAGGACTATAAGATACCAGCGTTTCCCCCTGGAGCTCCCTCGTGCGCTCTCCTGTTCCGACCCTGCCGCTTACCGGATACCTGTCCGCCTTTCTCCCTTTCGGGAGCGTGCGCTTTCTCATAGCTCACGCTGTAGTATTCCTCAGTTCGGTGTAAGTCGTCGCCTCCAGCTGGCTGTGTGCACGACCCCCGGTCAGCCCGGACCGCTGCCTATCCGGTACTATCGCTGGTTCCACCCGGTCAGACCAGACACTTATTCGCCCCCCCTG

5. gma-MIR-N1b,c,d

TTAAACGACCGATGTAGAAAG

>5-12-PJET1-2-forward_D03.ab1

TCCGACTCGATGGCTCGAGTTTTTCAGCAGATGCTGTCAACGATACGCTACGTAACGGCATGACAGTGTTTTTTTTTTTTCTTTCTACATCGGTCGTTTAAATCTTTCTAGAAGATCTCCTACAATATTCTCAGCTGCCATGGAAAATCGATGTTCTTCTTTTATTCTCTCAAGATTTTCAGGCTGTATATTAAAACTTATATTAAGAACTATGCTAACCACCTCATCAGGAACCGTTGTAGGTGGCGTGGGTTTTCTTGGCAATCGACTCTCATGAAAACTACGAGCTAAATATTCAATATGTTCCTCTTGACCAACTTTATTCTGCATTTTTTTTGAACGAGGTTTAGAGCAAGCTTCAGGAAACTGAGACAGGAATTTTATTAAAAATTTAAATTTTGAAGAAAGTTCAGGGTTAATAGCATCCATTTTTTGCTTTGCAAGTTCCTCAGCATTCTTAACAAAAGACGTCTCTTTTGACATGTTTAAAGTTTAAACCTCCTGTGTGAAATTATTATCCGCTCATAATTCCACACATTATACGAGCCGGAAGCATAAAGTGTAAAGCCTGGGGTGCCTAATGAGTGAGCTAACTCACATTAATTGCGTTGCGCTCACTGCCAATTGCTTTCCAGTCGGGAAACCTGTCGTGCCAGCTGCATTAATGAATCGGCCAACGCGCGGGGAGAGGCGGTTTGCGTATTGGGCGCTCTTCCGCTTCCTCGCTCACTGACTCGCTGCGCTCGGTCGTTCGGCTGCGGCGAGCGGTATCAGCTCACTCAAAGGCGGTAATACGGTTATCCACAGAATCAGGGGATAACGCAGGAAAGAACATGTGAGCAAAAGGCCAGCAAAAGGCCAGGAACCGTAAAAAGGCCGCGTTGCTGGCGTTTTTCCATAGGCTCCGCCCCCCTGACGAGCATCACAAAAATCGACGCTCAAGTCAGAGTGGCGAAACCCGACAGGACTATAAAGATACCAGCGTTTCCCCCTGGAAGCTCCCTCGTGCGCCTCTCCTGTTCCGACCCTGCCGCTTACCGGATACCTGTCCGCCTTTCTCCCTTCGGAAGCGTGCGCTTTCCTCATAGCTCACGCTGTAGTATCTCAGTCGTGTAGTCGTCGCTCCAGGCTTGGCTGTGTGCACGACCCCCCGTCAGCCCGGACGGCTGCGACTTATCAGTACTATCGACTGATCAACCCGGTAGGACCGGACTTATCCGCCACTGGGCCGAGCAAGCCCCACCTGTGATT

6. gma-MIR-N2

TAAAATATTAGACTGCTGTACT

>6-5-PJET1-2-forward_B06.ab1

CCGGATACTCGGATGGCTCGAGTTTTTCAGCAAGATTGTCAACGATACGCTACGTAACGGCATGACAGTGTTTTTTTTTTTTTTTTTTTTTTCCTGGAAGTACAGCAGTCTAATATTTTAGCATCTTTCTAGAAGATCTCCTACAATATTCTCAGCTGCCATGGAAAATCGATGTTCTTCTTTTATTCTCTCAAGATTTTCAGGCTGTATATTAAAACTTATATTAAGAACTATGCTAACCACCTCATCAGGAACCGTTGTAGGTGGCGTGGGTTTTCTTGGCAATCGACTCTCATGAAAACTACGAGCTAAATATTCAATATGTTCCTCTTGACCAACTTTATTCTGCATTTTTTTTGAACGAGGTTTAGAGCAAGCTTCAGGAAACTGAGACAGGAATTTTATTAAAAATTTAAATTTTGAAGAAAGTTCAGGGTTAATAGCATCCATTTTTTGCTTTGCAAGTTCCTCAGCATTCTTAACAAAAGACGTCTCTTTTGACATGTTTAAAGTTTAAACCTCCTGTGTGAAATTATTATCCGCTCATAATTCCACACATTATACGAGCCGGAAGCATAAAGTGTAAAGCCTGGGGTGCCTAATGAGTGAGCTAACTCACATTAATTGCGTTGCGCTCACTGCCAATTGCTTTCCAGTCGGGAAACCTGTCGTGCCAGCTGCATTAATGAATCGGCCAACGCGCGGGGAGAGGCGGTTTGCGTATTGGGCGCTCTTCCGCTTCCTCGCTCACTGACTCGCTGCGCTCGGTCGTTCGGCTGCGGCGAGCGGTATCAGCTCACTCAAAGGCGGTAATACGGTTATCCACAGAATCAGGGGATAACGCAGGAAAGAACATGTGAGCAAAAGGCCAGCAAAAGGCCAGGAACCGTAAAAAGGCCGCGTTGCTGGCGTTTTTCCATAGGCTCCGCCCCCCTGACGAGCATCACAAAAATCGACGCTCAAGTCAGAGGTGGCGAAACCCGACAGGACTATAAAGATACCAGGCGTTTCCCCTGGAAGCTCCCTCGTGCGCTCTCTGTCGACCCTGCGCTTACCGGATACCTGTCGCTTCTCCTCGGGAGCGTGGCGGCTTTCTCATAGCTCAGGCTGTAGTATCTCAGTCGGTGTAGTCGTTCGCTCAGCTGGACTGGTGACGAATCCCCCGGTCAGCGGACCGTGCCTATCGGTACCATCGGCTGATCACCGGGTAGAACCCGAACCTA

7. gma-MIR-N3

AACCGATGTAGAATGCTAGACA

>7-4-PJET1-2-reverse_D03.ab1

CCGGTTCATGTAGAGATCTTCTAGAAGATAACCGATGTAGAATGCTAGACAAAAAAAAAAAAAAAAAAAAACACTGTCATGCCGTTACGTAGCGTATCGTTGACAGCATCTTGCTGAAAAACTCGAGCCATCCGGAAGATCTGGCGGCCGCTCTCCCTATAGTGAGTCGTATTACGCCGGATGGATATGGTGTTCAGGCACAAGTGTTAAAGCAGTTGATTTTATTCACTATGATGAAAAAAACAATGAATGGAACCTGCTCCAAGTTAAAAATAGAGATAATACCGAAAACTCATCGAGTAGTAAGATTAGAGATAATACAACAATAAAAAAATGGTTTAGAACTTACTCACAGCGTGATGCTACTAATTGGGACAATTTTCCAGATGAAGTATCATCTAAGAATTTAAATGAAGAAGACTTCAGAGCTTTTGTTAAAAATTATTTGGCAAAAATAATATAATTCGGCTGCAGGGGCGGCCTCGTGATACGCCTATTTTTATAGGTTAATGTCATGATAATAATGGTTTCTTAGACGTCAGGTGGCACTTTTCGGGGAAATGTGCGCGGAACCCCTATTTGTTTATTTTTCTAAATACATTCAAATATGTATCCGCTCATGAGACAATAACCCTGATAAATGCTTCAATAATATTGAAAAAGGAAGAGTATGAGTATTCAACATTTCCGTGTCGCCCTTATTCCCTTTTTTGCGGCATTTTGCCTTCCTGTTTTTGCTCACCCAGAAACGCTGGTGAAAGTAAAAGATGCTGAAGATCAGTTGGGTGCACGAGTGGGTTACATCGAACTGGATCTCAACAGCGGTAAGATCCTTGAGAGTTTTCGCCCCGAAGAACGTTTTCCAATGATGAGCACTTTTAAAGTTCTGCTATGTGGCGCGGTATTATCCCGTATTGACGCCGGGCAAGAGCAACTCGGTCGCCGCATACACTATTCTCAGAATGACTTGGTTGAGTACTCACCAGTCACAGGAAAAGCATCTTACGGGATGGCATGACAGTAAGAGAATTATGCAGTGCTGCCATACCATGGAGTGATAACACTGCGGCAACTACTTCTGACACGATCGGAGGACCGAAGGAAGCTAACGCTTTTTTGCACAACATGGGGGGATCATGTACTCGGCCTTGATCGTTGGAACCGAGCTGAATGAAGCATACAACGACGAGCGTGAACCCGACGATGCTTAGCAATGGCACCAAGCTGTATGTCGCCAGAT

8. gma-MIR-N4

TCTTGACTTTGGACTTTTGGGT

>8-9-PJET1-2-forward_A05.ab1

CTCCCACTCAGATGGCTCGAGTTTTTCAGCAGATTCTTGACTTTGGACTTTTGGGTGAATTGTCGTATATTTTAATAAGCTGACTGGTGTGATAAAAAAAAAAAAAAAAAAAACACTGTCATGCCGTTACGTAGCGTATCGTTGACAATCTTTCTAGAAGATCTCCTACAATATTCTCAGCTGCCATGGAAAATCGATGTTCTTCTTTTATTCTCTCAAGATTTTCAGGCTGTATATTAAAACTTATATTAAGAACTATGCTAACCACCTCATCAGGAACCGTTGTAGGTGGCGTGGGTTTTCTTGGCAATCGACTCTCATGAAAACTACGAGCTAAATATTCAATATGTTCCTCTTGACCAACTTTATTCTGCATTTTTTTTGAACGAGGTTTAGAGCAAGCTTCAGGAAACTGAGACAGGAATTTTATTAAAAATTTAAATTTTGAAGAAAGTTCAGGGTTAATAGCATCCATTTTTTGCTTTGCAAGTTCCTCAGCATTCTTAACAAAAGACGTCTCTTTTGACATGTTTAAAGTTTAAACCTCCTGTGTGAAATTATTATCCGCTCATAATTCCACACATTATACGAGCCGGAAGCATAAAGTGTAAAGCCTGGGGTGCCTAATGAGTGAGCTAACTCACATTAATTGCGTTGCGCTCACTGCCAATTGCTTTCCAGTCGGGAAACCTGTCGTGCCAGCTGCATTAATGAATCGGCCAACGCGCGGGGAGAGGCGGTTTGCGTATTGGGCGCTCTTCCGCTTCCTCGCTCACTGACTCGCTGCGCTCGGTCGTTCGGCTGCGGCGAGCGGTATCAGCTCACTCAAAGGCGGTAATACGGTTATCCACAGAATCAGGGGATAACGCAGAAAGAACATGTGAGCAAAAGGCCAGCAAAAGGCCAGGAACCGTAAAAAGGCCGCGTTGCTGGCGTTTTTCCATAGGCTCCGCCCCCCTGACGAGCATCACAAAAATCGACGCTCAAGTCAGAGGTGGCGAAACCCGACAGGACTATAAGAATACCAGCGTTTCCCCCTGGAGCTCCCTCGTGCGCTCTCCTGTCGACCCTGCGCTTACGGATACTGTCGCTTCTCCTTTCGGGAGCGTGCGCTTTCTCATAGCTCACGCTGTAGTATTCTCAGTCGGTGTAGGTCGTTCGCTCCAGGCTTGGCTGTGTGACTGAACCCCGGTCAGTCGAACGCTGCGCCTTATTCGGTTAACATTATCGGCTCTTTGAGAG

9. gma-MIR-N5

GTTTGGATAGAGAATTTTAAA

>9-12-PJET1-2-forward_E05.ab1

TCGGAACTCGATGGCTCGAGTTTTTCAGCAGATGCTGTCAACGATACGCTACGTAACGGCATGACAGTGTTTTTTTTTTTTTTTTTTTTTTAAAATTCTCTATCCAAACGGGATCTTTCTAGAAGATCTCCTACAATATTCTCAGCTGCCATGGAAAATCGATGTTCTTCTTTTATTCTCTCAAGATTTTCAGGCTGTATATTAAAACTTATATTAAGAACTATGCTAACCACCTCATCAGGAACCGTTGTAGGTGGCGTGGGTTTTCTTGGCAATCGACTCTCATGAAAACTACGAGCTAAATATTCAATATGTTCCTCTTGACCAACTTTATTCTGCATTTTTTTTGAACGAGGTTTAGAGCAAGCTTCAGGAAACTGAGACAGGAATTTTATTAAAAATTTAAATTTTGAAGAAAGTTCAGGGTTAATAGCATCCATTTTTTGCTTTGCAAGTTCCTCAGCATTCTTAACAAAAGACGTCTCTTTTGACATGTTTAAAGTTTAAACCTCCTGTGTGAAATTATTATCCGCTCATAATTCCACACATTATACGAGCCGGAAGCATAAAGTGTAAAGCCTGGGGTGCCTAATGAGTGAGCTAACTCACATTAATTGCGTTGCGCTCACTGCCAATTGCTTTCCAGTCGGGAAACCTGTCGTGCCAGCTGCATTAATGAATCGGCCAACGCGCGGGGAGAGGCGGTTTGCGTATTGGGCGCTCTTCCGCTTCCTCGCTCACTGACTCGCTGCGCTCGGTCGTTCGGCTGCGGCGAGCGGTATCAGCTCACTCAAAGGCGGTAATACGGTTATCCACAGAATCAGGGGATAACGCAGGAAAGAACATGTGAGCAAAAGGCCAGCAAAAGGCCAGGAACCGTAAAAAGGCCGCGTTGCTGGCGTTTTTCCATAGGCTCCGCCCCCCTGACGAGCATCACAAAAATCGACGCTCAAGTCAGAGGTGGCGAAACCCGACAGGACTATAAAGATACCAGCGTTTCCCCCTGGAAGCTCCCTCGTGCGCTCTCCTGTTCCGACCCTGCGCTTACGGGATACCTGTCCGCCTTTCTCCTTCGGAGCGTGCGCTTTCTCATAGCTCACGCTGTAAGTATCTCAGTCGGTGTAGTCGGTCGCTTCAGCCTGGGCCTGTGTGCACGACCCCGGTCAGGTGGAACGGCTGCGCTACGTACTATCGACTTGATCATCGTAGAACACGAACTTATTCGCCCACTGGCAGGCA

10. gma-MIR-N6

CACTTTGGATTTGATATACTT

>10-1-PJET1-2-forward_D07.ab1

TTCGGAACTCGATGGCTCGAGTTTTCAGCAGATGGGCACTTTGGATTTGATATACTTAAAAAAAAAAAAAAAAAAAAACACTGTCATGCCGTTACGTAGCGTATCGTTGACAGCATCTTTCTAGAAGATCTCCTACAATATTCTCAGCTGCCATGGAAAATCGATGTTCTTCTTTTATTCTCTCAAGATTTTCAGGCTGTATATTAAAACTTATATTAAGAACTATGCTAACCACCTCATCAGGAACCGTTGTAGGTGGCGTGGGTTTTCTTGGCAATCGACTCTCATGAAAACTACGAGCTAAATATTCAATATGTTCCTCTTGACCAACTTTATTCTGCATTTTTTTTGAACGAGGTTTAGAGCAAGCTTCAGGAAACTGAGACAGGAATTTTATTAAAAATTTAAATTTTGAAGAAAGTTCAGGGTTAATAGCATCCATTTTTTGCTTTGCAAGTTCCTCAGCATTCTTAACAAAAGACGTCTCTTTTGACATGTTTAAAGTTTAAACCTCCTGTGTGAAATTATTATCCGCTCATAATTCCACACATTATACGAGCCGGAAGCATAAAGTGTAAAGCCTGGGGTGCCTAATGAGTGAGCTAACTCACATTAATTGCGTTGCGCTCACTGCCAATTGCTTTCCAGTCGGGAAACCTGTCGTGCCAGCTGCATTAATGAATCGGCCAACGCGCGGGGAGAGGCGGTTTGCGTATTGGGCGCTCTTCCGCTTCCTCGCTCACTGACTCGCTGCGCTCGGTCGTTCGGCTGCGGCGAGCGGTATCAGCTCACTCAAAGGCGGTAATACGGTTATCCACAGAATCAGGGGATAACGCAGGAAAGAACATGTGAGCAAAAGGCCAGCAAAAGGCCAGGAACCGTAAAAAGGCCGCGTTGCTGGCGTTTTTCCATAGGCTCCGCCCCCCTGACGAGCATCACAAAAATCGACGCTCAAGTCAGAGTGGCGAAACCCGACAGGACTATAAAGATACCAGGCGTTTCCCCCTGGAGCTCCCTCGTGCGCTCTCTGTCGACCTGCGCTACGGATACTGTCGCTTTCTCCTCGGGAGCGTGCGCTTTCTCATAGCTCACGCTGTAGTATCTCAGTCGGTGTAGTTCGTCGCTTCAGGCCTGGGCCTGGTGTGCACGGACCCCCCGGTTCAGGTCGAACGGCTGGCCTATCCGGTACATCGCTGGATCAACGTAGGACGACTATTCGCCACTGGGCGAGAGAGTCATG

11. gma-MIR-N7

AACACAATGGAATCGTGATTTCG

>11-23-PJET1-2-forward_B07.ab1

TCCCCACTCCGATGCTCGAGTTTTTCAGCAAGATACGATACGCTACGTAACGGCATGACAGCGTTTTTTTTTTTTTTTTTTTTCTTCGGGCGAAATCACGATTCCATTGTGTTATCTTTCTAGAAGATCTCCTACAATATTCTCAGCTGCCATGGAAAATCGATGTTCTTCTTTTATTCTCTCAAGATTTTCAGGCTGTATATTAAAACTTATATTAAGAACTATGCTAACCACCTCATCAGGAACCGTTGTAGGTGGCGTGGGTTTTCTTGGCAATCGACTCTCATGAAAACTACGAGCTAAATATTCAATATGTTCCTCTTGACCAACTTTATTCTGCATTTTTTTTGAACGAGGTTTAGAGCAAGCTTCAGGAAACTGAGACAGGAATTTTATTAAAAATTTAAATTTTGAAGAAAGTTCAGGGTTAATAGCATCCATTTTTTGCTTTGCAAGTTCCTCAGCATTCTTAACAAAAGACGTCTCTTTTGACATGTTTAAAGTTTAAACCTCCTGTGTGAAATTATTATCCGCTCATAATTCCACACATTATACGAGCCGGAAGCATAAAGTGTAAAGCCTGGGGTGCCTAATGAGTGAGCTAACTCACATTAATTGCGTTGCGCTCACTGCCAATTGCTTTCCAGTCGGGAAACCTGTCGTGCCAGCTGCATTAATGAATCGGCCAACGCGCGGGGAGAGGCGGTTTGCGTATTGGGCGCTCTTCCGCTTCCTCGCTCACTGACTCGCTGCGCTCGGTCGTTCGGCTGCGGCGAGCGGTATCAGCTCACTCAAAGGCGGTAATACGGTTATCCACAGAATCAGGGGATAACGCAGGAAAGAACATGTGAGCAAAAGGCCAGCAAAAGGCCAGGAACCGTAAAAAGGCCGCGTTGCTGGCGTTTTTCCATAGGCTCCGCCCCCCTGACGAGCATCACAAAAATCGACGCTCAAGTCAGAGGTGGCGAAACCCGACAGGACTATAAGATACCAGGCGTTTCCCCCTGGAAGCTCCCTCGTGCGCTCTCCTGTCCGACCTGCGCTTACGGATACCTGTCCGCTTTCTCCTTCGGAGCGTGCGCTTTCTCATAGCTCACGCTGTAGTATCTCAGTCGGTGTAAGTCGGTCGCTCAGCTGGCCTGTGTGCACGACCCCGGTCAGTGGACGCTGCGCCTTATCGGTACTATTCGTCTTGAGTTCACTGGTAGAACGGACTTATCGCCAACTGTGGCGAGCAGC

12. gma-MIR-N8

GTTTTGATATCACTGTCCAAAG

>12-3-PJET1-2-forward_C01.ab1

GTCGGAACTCGGATGGCTCGAGTTTTTCAGCAAGATGTTTTGATATCACTGTCCAAAGAAAAAAAAAAAAAAAAACACTGTCATGCCGTTACGTAGCGTATCGTTGACAGATCTTTCTAGAAGATCTCCTACAATATTCTCAGCTGCCATGGAAAATCGATGTTCTTCTTTTATTCTCTCAAGATTTTCAGGCTGTATATTAAAACTTATATTAAGAACTATGCTAACCACCTCATCAGGAACCGTTGTAGGTGGCGTGGGTTTTCTTGGCAATCGACTCTCATGAAAACTACGAGCTAAATATTCAATATGTTCCTCTTGACCAACTTTATTCTGCATTTTTTTTGAACGAGGTTTAGAGCAAGCTTCAGGAAACTGAGACAGGAATTTTATTAAAAATTTAAATTTTGAAGAAAGTTCAGGGTTAATAGCATCCATTTTTTGCTTTGCAAGTTCCTCAGCATTCTTAACAAAAGACGTCTCTTTTGACATGTTTAAAGTTTAAACCTCCTGTGTGAAATTATTATCCGCTCATAATTCCACACATTATACGAGCCGGAAGCATAAAGTGTAAAGCCTGGGGTGCCTAATGAGTGAGCTAACTCACATTAATTGCGTTGCGCTCACTGCCAATTGCTTTCCAGTCGGGAAACCTGTCGTGCCAGCTGCATTAATGAATCGGCCAACGCGCGGGGAGAGGCGGTTTGCGTATTGGGCGCTCTTCCGCTTCCTCGCTCACTGACTCGCTGCGCTCGGTCGTTCGGCTGCGGCGAGCGGTATCAGCTCACTCAAAGGCGGTAATACGGTTATCCACAGAATCAGGGGATAACGCAGGAAAGAACATGTGAGCAAAAGGCCAGCAAAAGGCCAGGAACCGTAAAAAGGCCGCGTTGCTGGCGTTTTTCCATAGGCTCCGCCCCCCTGACGAGCATCACAAAATCGACGCTCAAGTCAGAGGTGGCGAAACCCGACAGGACTATAAAGATACCAGGCGTTTCCCCCTGGAAAGCTCCCTCGTGCGCTCTCCTGTTCCGACCCTGCCGCTTACCGGATACCTGTCCGCCTTTCTCCCTTCGGAGCGTGCGCTTTCTCATAGCCTCACGCTGTAGTATCTCAGTCCGGTGTAGTCGTCGCCTCCAAGCTTGGCTGTGTGCACGATCCCCCGTCAGCGACGGCTGCGACGTTATCGGATAACTATCGACTGGATCAACCGGGTTAGAACCGAACTTATCGCCAACTTGGGAGCACGATCCACCATGAGTA
