# Supplementary material for: Comparative Profiling of microRNA Expression in Soybean Seeds from Genetically Modified Plants and their Near-Isogenic Parental Lines
Source: PLoS One. 2016 May 23;11(5):e0155896. doi: 10.1371/journal.pone.0155896 (PMC4876996; doi:10.1371/journal.pone.0155896)
Supplement: S2 Table — (DOCX) [file pone.0155896.s007.docx]

**Supplemental File:**

**Table S2.** Target prediction of novel gma-miRNAs using psRNATarget online.

Paper title: "Comparative profiling of microRNA expression in soybean seeds from genetically modified plants and their near-isogenic parental lines"

Author: Yong Wang, Qingkuo Lan, Xin Zhao, Wentao Xu, Feiwu Li, Qinying Wang*, Rui Chen*

Date: Mar. 2016

Contact: chenrui.2011@outlook.com

**Table S2.** Target prediction of novel gma-miRNAs using psRNATarget online.

| **miRNAs** | **Targets** | **Exp** | **UPE** | **miRNA**  **start..end** | **Target**  **start..end** | **miRNA_aligned_fragment** | **Target_aligned_fragment** | **Inhibition** | **Target_Desc.** |
| --- | --- | --- | --- | --- | --- | --- | --- | --- | --- |
| gma-miR1516-N1 | TC455346 | 2 | 16 | 1..22 | 373..394 | AUAUAUUUUCUGUAGAGAAGCU | AACUUCUCAACUGAAAAUAUAU | Translation | None |
| gma-miR4401-N1 | BM567682 | 2 | 15 | 1..20 | 147..166 | AAAGACGUUGUUGAGGUAAG | UUUAUUUCAACAACGUUUUU | Cleavage | UniRef100_Q8I1D7 Cluster |
| gma-miR-N4 | TC460481 | 2 | 19 | 1..20 | 66..85 | UCUUGACUUUGGACUUUUGG | CUAAAAGUCCAAAGUAAAGA | Cleavage | similar to UniRef100_A2Q3V4 Cluster: Integral membrane protein DUF6 containing protein |
| gma-miR-N5 | TC428527 | 2 | 11 | 1..21 | 121..141 | UAUGUUUGGAUAGAGAAUUUU | AAGAUUCUCUAACCAAAUAUA | Translation | similar to UniRef100_Q0GPH2 Cluster: BZIP transcription factor |
| gma-miR-N5 | TC460685 | 2 | 5 | 1..21 | 196..216 | UAUGUUUGGAUAGAGAAUUUU | AAGAUUCUCUAACCAAAUAUA | Translation | similar to UniRef100_Q0GPH3 Cluster: BZIP transcription factor |
| gma-miR-N7 | TC428298 | 2 | 12 | 1..24 | 704..727 | AACACAAUGGAAUCGUGAUUUCGU | AUUGAAUCAUGAUUUUGUUGUGUU | Cleavage | None |

Parameters of psRNATarget: Maximum expectation = 2.0; Glycine max (soybean), unigene, DFCI Gene Index (GMGI, version 16).
