# Supplementary material for: Comparative Profiling of microRNA Expression in Soybean Seeds from Genetically Modified Plants and their Near-Isogenic Parental Lines
Source: PLoS One. 2016 May 23;11(5):e0155896. doi: 10.1371/journal.pone.0155896 (PMC4876996; doi:10.1371/journal.pone.0155896)
Supplement: S3 Table — (DOCX) [file pone.0155896.s008.docx]

**Supplemental File:**

**Table S3.** Primers used for qRT-PCR and RT-PCR experiments.

Paper title: "Comparative profiling of microRNA expression in soybean seeds from genetically modified plants and their near-isogenic parental lines"

Author: Yong Wang, Qingkuo Lan, Xin Zhao, Wentao Xu, Feiwu Li, Qinying Wang*, Rui Chen*

Date: Mar. 2016

Contact: chenrui.2011@outlook.com

**Table S3.** Primers used for qRT-PCR and RT-PCR experiments in this study.

| ID |  | **Primer sequence(5’-3’)** | **Length (nt)** |
| --- | --- | --- | --- |
| qRT-PCR | gma-miR1507a | GGTCTCATTCCATACATCGTCTG | 23 |
|  | gma-miR1510b-5p | CCAGGGATAGGTAAAACAACTAC | 23 |
|  | gma-miR1511 | GCAACCAGGCTCTGATACCATG | 22 |
|  | gma-miR166a-3p;h-3p;u | TCTCGGACCAGGCTTCATTC | 20 |
|  | gma-miR319a | AATTGGACTGAAGGGAGCTCC | 21 |
|  | gma-miR390a-5p;e | AAGCTCAGGAGGGATAGCG | 19 |
|  | gma-miR396b-5p | GGTTCCACAGCTTTCTTGAAC | 21 |
|  | gma-miR398c | AATGTGTTCTCAGGTCGCCCCTG | 23 |
|  | gma-miR482b | TCTTCCCTACACCTCCCATAC | 21 |
|  | gma-miR482c | TTCCCAATTCCGCCCATTCC | 20 |
|  | gma-U6 | ACGCACAAATCGAGAAATGGTC | 22 |
| RT-PCR | gma-miR1516-N1 | CCATATATTTTCTGTAGAGAAGC | 23 |
|  | gma-miR4374-N1 | GTAAGCACCGTCTTTGAATGCC | 22 |
|  | gma-miR4401-N1 | AGACGTTGTTGAGGTAAGCACC | 22 |
|  | gma-miR-N1a | GCATAACCGATCTAGAATGTAAT | 23 |
|  | gma-miR-N1b,c,d | CCTTAAACGACCGATGTAGAAAG | 23 |
|  | gma-miR-N2a | GCTAAAATATTAGACTGCTGTAC | 23 |
|  | gma-miR-N3a | AACCGATGTAGAATGCTAGAC | 21 |
|  | gma-miR-N4a | TCTTGACTTTGGACTTTTGGG | 21 |
|  | gma-miR-N5a | CCCGTTTGGATAGAGAATTTTAAA | 24 |
|  | gma-miR-N6a | GGGCACTTTGGATTTGATATACTT | 24 |
|  | gma-miR-N7a | AACACAATGGAATCGTGATTTCG | 23 |
|  | gma-miR-N8a | GTTTTGATATCACTGTCCAAAG | 22 |
| Reverse primer | | GCTGTCAACGATACGCTACGTAACGGCATGACAGTGT(24)VN | 62 |
| Downstream universal primer | | GCTGTCAACGATACGCTACGTAACG | 25 |
